# Decoding the mechanistic basis of liver–muscle communication in health and disease

**DOI:** 10.1007/s00210-026-05015-3

**Published:** 2026-02-19

**Authors:** Noha M. Gamil, Habiba A. Elsayed, Esraa T. Salah, Heba A. Mostafa, Riham A. El-Shiekh, Heba R. Ghaiad, Hebatollah E. Eitah

**Affiliations:** 1 Department of Pharmacology and Toxicology, College of Pharmaceutical Sciences and Drug Manufacturing, Misr University for Science and Technology (MUST), P.O. Box 77, Giza, Egypt; 2 College of Pharmaceutical Sciences and Drug Manufacturing, Misr University for Science and Technology (MUST), P.O. Box 77, Giza, Egypt; 3 Department of Pharmacognosy, Faculty of Pharmacy, Cairo University, Cairo, 11562 Egypt; 4 Department of Biochemistry, Faculty of Pharmacy, Cairo University, Cairo, 11562 Egypt; 5 Medicinal and Pharmaceutical Chemistry Department, Pharmacology Group, National Research Centre, Dokki, Giza, Egypt

**Keywords:** Hepatokines, Liver–muscle axis, Liver fibrosis, MASLD, Myokines, Sarcopenia

## Abstract

The bidirectional communication between liver and skeletal muscle represents a critical yet underexplored axis in human physiology. Dysfunction in either organ can accelerate pathology in the other, amplifying disease progression. Understanding this interconnected system is essential for developing targeted and effective therapeutic strategies. This comprehensive review elucidates the complex pathophysiological mechanisms underlying liver–muscle crosstalk and identifies novel therapeutic targets for simultaneous intervention in both organs. We analyzed peer-reviewed literature focusing on molecular pathways, biomarkers, and therapeutic interventions targeting the liver–muscle axis, including cardiac muscle interactions. Key parameters examined included inflammatory mediators (TNF-α, IL-6), metabolic regulators (mTOR, AMPK), hepatokines, myokines, cardiokines, and emerging biomarkers such as zonulin. The liver–muscle axis operates through multiple interconnected pathways: (1) inflammatory cascades where TNF-α inhibits muscle mTOR signaling while promoting hepatic stellate cell activation; (2) metabolic disruption through insulin resistance and AMPK pathway dysfunction affecting both organs simultaneously; (3) gut–liver–muscle crosstalk mediated by microbiome-derived metabolites and intestinal permeability markers like zonulin; (4) hepatokine–myokine signaling networks that coordinate metabolic homeostasis; and (5) liver–heart crosstalk involving cardiomyocyte–hepatocyte interactions through FGF21, IL-6/STAT3 signaling, and inflammatory pathways that distinguish cardiac muscle from skeletal muscle responses. Studying the liver–muscle axis helps in understanding metabolic diseases, transforming them from isolated organ pathologies to interconnected systemic disorders. This framework opens new avenues for precision medicine approaches, biomarker development, and therapeutic innovation that simultaneously optimize liver, skeletal muscle, and cardiac health.

## Introduction

The liver–muscle axis serves as a crucial framework linking liver health and muscle function, both of which are essential for metabolic regulation. The liver regulates glucose production, lipid metabolism, and protein synthesis, while maintaining energy balance and releasing hormones and cytokines that modulate muscle function (Jiang et al. 2025). Skeletal muscle serves as the primary reservoir for glucose disposal and glycogen deposition, and it is integrally involved in regulating protein turnover. Disruption of this interactive network can foster a spectrum of pathological states, including sarcopenia, non-alcoholic fatty liver disease (NAFLD), non-alcoholic steatohepatitis (NASH), and, in an advanced stage, liver cirrhosis (Wong and Yuan 2024).

NAFLD is closely linked to skeletal muscle dysfunction. Excess hepatic lipid accumulation promotes peripheral insulin resistance and low-grade inflammation, both of which impair muscle protein metabolism and favor catabolism. The term NAFLD is no longer used, as it has been replaced by MASLD or metabolic dysfunction-associated steatotic liver disease. Individuals with MASLD typically present with diminished muscle mass and strength, a complication that further exacerbates their overarching metabolic derangement (De Bandt et al. 2018). The progressive inflammatory form of MASLD was previously referred to as NASH and is now termed MASH (metabolic dysfunction-associated steatohepatitis), which further exacerbates muscle wasting through intensified hepatic inflammation, oxidative stress, and fibrosis. The situation becomes acute in cirrhosis, where liver function is progressively lost, and the hepatic milieu becomes hostile to both hepatic and extrahepatic tissues. In cirrhosis, the liver’s capacity to produce key proteins and hormones needed to preserve skeletal muscle wanes. This deterioration sets the stage for protein-energy malnutrition and the gradual yet relentless loss of muscle mass termed sarcopenia, which together diminishes prognosis and quality of life (Nishikawa et al. 2024).

Sarcopenia, characterized by the progressive loss of muscle quantity and functional capacity that accompanies advancing age, is exacerbated by concurrent liver disease. This is attributable to the liver’s diminished capacity to produce the structural proteins and anabolic mediators, such as insulin-like growth factors-1 (IGF-1) and angiotensin II, that are essential for muscle remodeling, repair, and hypertrophic stimuli (Nishikawa et al. 2024). Emerging epidemiological data reveal the escalating toll of sarcopenia in liver disease. In MASLD, the condition can be found, depending on the cohort, in 1.6% to 63.0% of patients, with the bulk of investigations exceeding 10.0% (Giri et al. 2024). Among cirrhotic patients, estimates cluster around 41% on average, with disparities determined by diagnostic methods and study demographics (Cui et al. 2024). These divergent figures argue for uniform diagnostic protocols and, more critically, draw attention to the pervasive and alarming loss of muscle in liver disease, which imperils the patient’s future.

Clinically, the implications of sarcopenia within this group are grave. The loss of skeletal muscle serves as an independent catalyst for more rapid liver deterioration, accelerating the liver’s progression toward decompensation (Leunis et al. 2023). Patients harboring sarcopenia and liver disease face an elevated burden of complications, including hepatic encephalopathy, sepsis, and more extended hospital stays (Chang et al. 2019). The most sobering comparison is mortality: cirrhotic patients who also display muscle wasting can expect unambiguously shorter survival than their counterparts who do not (Topan et al. 2021).

Integrating these epidemiological findings highlights the pressing need to address liver–muscle interactions in public health practice. The widespread occurrence and harmful impact of sarcopenia on the trajectory of liver disease warrant that clinicians routinely assess muscle mass alongside liver function. By identifying and addressing muscle wasting in its earliest stages, healthcare teams can initiate tailored nutritional support, structured physical rehabilitation, and emerging pharmacological strategies that collectively slow disease progression and elevate patients’ functional status.

This review seeks to refine our understanding of the liver–muscle axis by elucidating the underlying pathophysiological mechanisms common to liver and muscle disorders. A more precise understanding of these interrelated processes will inform the design of targeted interventions, including dietary optimization, tailored exercise programs, and specific drug combinations, capable of counteracting the deleterious spiral linking liver and muscle impairment. Such proactive measures aim not only to preserve muscle integrity and metabolic health but also to enhance the overall effectiveness of therapies directed at metabolic and liver diseases, ultimately resulting in better long-term outcomes for affected individuals.

## Review methodology

A comprehensive literature search was conducted to identify relevant studies exploring the liver–muscle axis and its role in metabolic regulation. Relevant studies published between January 2008 and December 2025 were identified by searching PubMed, Scopus, and Web of Science using keywords including “liver-muscle axis,” “liver fibrosis,” “muscle atrophy,” and “liver fatty disease”. Studies were included in this review if they were peer-reviewed articles investigating the physiological or biochemical interactions between the liver and skeletal muscle. Only original research articles and comprehensive reviews published in English were considered.

Data extraction focused on key parameters relevant to liver–muscle axis, including study design (e.g., clinical trials and animal models), population characteristics (e.g., age, sex, and health status), interventional details (e.g., type of exercise and dietary interventions), and outcome measures (e.g., insulin sensitivity, muscle protein synthesis, and liver enzyme levels).

## Anatomy of liver–muscle axis: mechanisms underlying interorgan communication

The liver is the body’s heaviest gland, nestled in the upper right quadrant of the abdomen and tipping the scales at roughly 1.5 kg. It is organized into eight metabolic segments, each receiving a dedicated blood supply and having its own bile duct system. These segments group into the right, left, quadrate, and caudate lobes, collectively undergirding the liver’s multifaceted activities. This organ handles detoxifying agents, synthesizing plasma proteins, and generating bile salts and other molecules crucial for digestion. Blood reaches the liver from two parallel courses: the oxygen-rich hepatic artery and the portal vein, which channels some of the freshly absorbed nutrients from the gastrointestinal tract, thereby coupling nutrient acquisition with the critical task of detoxifying (Mahadevan 2020).

Skeletal muscle is a highly responsive tissue that drives every movement and helps maintain upright posture. Its cells are wrapped in the sarcolemma and packed with myofibrils, whose finely striped actin and myosin filaments slide past one another in a repeating segment called the sarcomere, resulting in the visible striations of the muscle belly. Muscle fibers are classified into three types: Type I fibers contract slowly and rely primarily on oxygen, Type IIa fibers switch between aerobic and anaerobic metabolism for speed and endurance, and Type IIb fibers rely mostly on anaerobic energy for rapid, powerful bursts of force (Mukund and Subramaniam 2020).

The liver and skeletal muscle are in constant, dynamic dialogue. When the muscles contract, they secrete signaling proteins, known as myokines, including interleukin-6 (IL-6) and fibroblast growth factor 21 (FGF21), which modulate liver function. IL-6, for instance, helps dampen the flames of liver inflammation and protect against MASLD. In the fasting state or during exercise, skeletal muscle releases alanine and lactate into the bloodstream. The liver uses these molecules, along with glycerol from fat breakdown, to manufacture new glucose in gluconeogenesis, a critical process that guards against drops in blood sugar when dietary carbohydrate is low (Romijn and Pijl 2009; Pacifico et al. 2019).

Skeletal muscle serves as a key reservoir for insulin-stimulated glucose disposal. Greater muscle volume enhances whole-body insulin sensitivity, thereby safeguarding the liver by attenuating hepatic lipid overload and reducing inflammatory signals. When liver–muscle communication falters, disorders like MASLD become more likely, especially when tandems of low muscle mass and disease manifestation are noted. Deterioration of muscle tissue aggravates insulin insensitivity, triggering elevated hepatic lipid accumulation and fostering a self-perpetuating cycle of muscle and liver decline (Pacifico et al. 2019).

## Biochemical basis: understanding the molecular mechanisms of liver fibrosis and muscle atrophy

Liver fibrosis and muscle atrophy are significant pathological conditions. Both involve intricate biochemical mechanisms governing cellular responses to stress, inflammation, and injury. Liver fibrosis originates from a complex interplay of cellular activation, inflammatory responses, extracellular matrix (ECM) remodeling, and genetic factors, characterized by excessive ECM accumulation primarily of collagen in response to chronic liver disease. Key events include activation of hepatic stellate cells (HSCs) into fibrogenic myofibroblasts, triggered by inflammatory cytokines (e.g., TNF-α and IL-6) released by hepatocytes and Kupffer cells (ZhangLiu and Yang, 2023). This activation occurs in response to liver damage caused by variables such as alcohol abuse, viral infections, and metabolic disorders (Addissouky et al. 2024). ECM components are produced by activated HSCs, including fibrillar collagens and tissue inhibitors of metalloproteinases (TIMPs). Chronic inflammation drives fibrosis progression by recruiting leukocytes and secreting profibrogenic cytokines. Stimuli, such as transforming growth factor-beta (TGF-β), activate transcription factors (e.g., Snail and Zeb), inducing hepatocytes to undergo an epithelial-mesenchymal transition (EMT) into invasive fibroblast-like cells (Zhang et al. 2023a, b). Antifibrotic therapies may target HSC activation, inflammation, EMT, oxidative stress, and ECM balance.

Muscle atrophy, characterized by loss of muscle mass and strength, often results from disuse, aging, or chronic diseases. Its mechanisms involve an imbalance between protein synthesis and degradation. The ubiquitin–proteasome pathway is pivotal in muscle protein breakdown, as the proteasome degrades ubiquitin-tagged proteins, leading to the loss of contractile proteins. During atrophy, FOXO transcription factors are upregulated, leading to increased expression of atrophy-related genes (atrogenes) that enhance proteolysis. Autophagy is also activated, facilitating the degradation of damaged organelles and proteins regulated by pathways like mammalian target of rapamycin (Mtor), which inhibits autophagy under nutrition-rich conditions but permits it during stress or nutrient scarcity (Sartori et al. 2021). Hormonal factors play a significant role; myostatin, a key inhibitor of muscle growth, activates SMAD2/3 signaling, promoting protein breakdown and reducing muscle hypertrophy, while glucocorticoids and inflammatory cytokines further accelerate muscle loss by enhancing proteolysis (Sartori et al. 2009).

## Interconnections between liver fibrosis and muscle atrophy

Liver fibrosis and skeletal muscle atrophy emerge from overlapping biochemical pathways characterized by persistent inflammation and adaptive cellular stress responses. In chronic liver disease, systemic inflammation fosters muscle protein breakdown through cytokine-mediated pathways. Nutritional factors further aggravate this relationship. Liver dysfunction disrupts hepatic metabolism of proteins, lipids, and carbohydrates, instigating a cycle of malnutrition that accelerates muscle wasting (Anand 2017). Conversely, loss of muscle mass becomes a determinant of frailty, complicating liver disease progression and recovery by impairing functional reserve and nutrient delivery to the liver (Bodine et al. 2023).

## Crosstalk between liver and muscles: bidirectional signaling pathways

The liver and skeletal muscle communicate through a network of dueling signaling pathways that govern systemic energy balance, particularly in pathologies such as MASLD and MASH. Elucidating this metabolic axis is crucial for understanding the pathogenesis of liver fibrosis and its associated muscle dysfunction.

Muscle influences hepatic function through the release of myokines that fine-tune liver metabolism. Follistatin-like protein 1 (FSTL1) serves as a prototypical myokine in this dialogue; its hepatic levels correlate positively with the severity of inflammation and fibrosis in experimental MASH. The myokine is transcriptionally regulated by the muscle-specific interferon regulatory factor 4 (IRF4). Genetic inactivation of IRF4 in skeletal muscle results in diminished circulating FSTL1 and attenuated hepatic steatosis and inflammation, demonstrating that the muscle-to-liver axis, via the IRF4-FSTL1 relay, has a substantive bearing on liver disease progression (Guo et al. 2023).

The liver releases several hepatokines, including hepatocyte nuclear factor 4-alpha (HNF4α), fetuin-A, and FGF21, that collectively enhance insulin sensitivity in skeletal muscle and promote the oxidation of muscle lipid stores, thereby contributing to the regulation of energy homeostasis. The liver also regulates glucose and lipid metabolism, directly impacting muscle function. In MASLD, insulin resistance disrupts muscle metabolism, contributing to sarcopenia and myosteatosis (Altajar and Baffy 2020). Additionally, liver dysfunction or malnutrition reduces muscle protein synthesis, worsening sarcopenia. Beyond the liver–muscle axis, other tissues or catabolic conditions may interact with these organs through direct or inflammation-mediated mechanisms, with bidirectional dynamics shifting across liver disease stages (Henin et al. 2022).

## Dietary influences on liver fibrosis: impact of macronutrients and micronutrients

A balanced diet is vital for maintaining liver function and preventing damage or disease progression. The liver, essential for digestion and toxin removal, can develop scar tissue from excessive ECM protein accumulation. Macronutrients (carbohydrates, proteins, and fats) and micronutrients (vitamins, minerals) can either exacerbate or alleviate liver fibrosis (Guveli et al. 2021).

High-quality proteins, such as those from fish proteins (e.g., salmon and mackerel), are rich in omega-3 fatty acids (Al-Attar and Al-Rethea 2017) and plant sources (e.g., soy and legumes) high in glutamate and glycine, enhance glutathione synthesis, reduce oxidative stress, and inhibit HSC activation (Khazaei et al. 2023), protecting against liver fibrosis. A high-protein diet may reverse MASLD, but the protein source and liver function must be considered (De Chiara et al. 2019). Fat intake significantly affects liver fibrosis: diets high in saturated fatty acids (SFA), trans fatty acids (TFA), and cholesterol promote fibrosis progression, while omega-3 fatty acids from fatty fish, flaxseeds, and walnuts offer protective effects by reducing inflammation and improving lipid metabolism (Jia et al. 2021). Carbohydrate type and source influence liver fat accumulation and promote de novo lipogenesis (DNL), thereby contributing to hepatic fat accumulation. In contrast, healthy carbohydrate sources, such as whole grains, fruits, and vegetables, provide healthy carbohydrates that support liver health (Kelly et al. 2023).

Micronutrients play a crucial role in metabolic processes. Deficiencies in folic acid, vitamin D, magnesium, zinc, and selenium are associated with increased disease risk. These nutrients possess antioxidant properties that reduce hepatic fat accumulation, potentially delaying the progression of MASLD to fibrosis or cirrhosis. Vitamin D levels inversely correlate with chronic liver disease severity due to their immunomodulatory and antifibrotic effects, with sources including fatty fish, fortified milk, and orange juice (Bertol et al. 2020). Magnesium deficiency promotes oxidative stress and elevated serum transaminases, which can worsen fibrosis, and is found in foods such as pineapple, pecans, and peanuts. Zinc and selenium, present in oysters, crabs, chickpeas, beef, pork, and other foods, support the antioxidant enzyme function, protecting against oxidative stress and liver injury (Nicoll et al. 2022).

## Exercise and muscle health: mitigating atrophy in liver dysfunction

The relationship between exercise, muscle health, and liver dysfunction is increasingly recognized as vital for improving outcomes in liver disease. Regular physical exercise enhances muscular strength and mass while mitigating the adverse effects of liver dysfunction, particularly in cirrhosis and MASLD. Exercise increases blood flow to the liver, reduces hepatic fat accumulation, and decreases inflammation, improving liver function and potentially reversing MASLD (Stine et al. 2021). It also helps maintain muscle mass and strength, counteracting sarcopenia, a frequent complication in liver dysfunction (Pacifico et al. 2019).

Exercise benefits both muscle and liver health by modulating metabolic pathways that affect these organs. It enhances insulin sensitivity and reduces hepatic fat formation (Romijn and Pijl 2009), and stimulates the release of myokines, which protect against liver inflammation and fat accumulation (Pacifico et al. 2019). Patients with liver dysfunction are encouraged to engage in at least 150 min of moderate-intensity aerobic exercise per week (e.g., brisk walking, swimming, and cycling) and incorporate resistance training twice a week to maintain muscle mass and counteract atrophy (Thorp and Stine 2020). Patients should begin slowly, increasing intensity gradually (e.g., by no more than 10% per week) to avoid strain or injury (Tandon et al. 2018). Consultation with healthcare professionals is essential before starting exercise regimens, particularly for those with severe liver disease or complications, to tailor programs to individual needs (Thorp and Stine 2020).

## Fatty liver disease and muscle atrophy: shared etiologies and pathogenic pathways

Fatty liver disease (FLD), particularly MASLD, and muscle atrophy share common etiologies and pathogenic pathways, including insulin resistance, obesity, chronic inflammation, and physical inactivity (Kim and Choi 2019). Understanding these interconnections is essential for developing effective therapies.

## Insulin resistance and obesity

Insulin resistance and obesity are central to both FLD and muscle atrophy. In skeletal muscle, IR activates the mammalian target of rapamycin complex 1 (mTORC1) and increases signaling to its downstream effectors 4E-binding protein 1 (4E-BP1) and ribosomal S6 kinase 1 (S6K1), resulting in muscle mass maintenance and skeletal muscle anabolism (Fry et al. 2011). Additionally, IR impairs glucose uptake and glycogen synthesis, increasing muscle breakdown and reducing mitochondrial function, which exacerbates hepatic IR and promotes MASLD via de novo lipogenesis (Kim and Choi 2019). Obesity amplifies this through the secretion of inflammatory cytokines and adipokines, such as leptin, from visceral adipose tissue, thereby fostering chronic inflammation and IR. Leptin upregulates proinflammatory cytokines and impairs the growth hormone/insulin-like growth factor-1 (GH/IGF-1) axis, reducing muscle protein synthesis (Cifuentes et al. 2025).

## Chronic low-grade inflammation

**Fig. 1:**
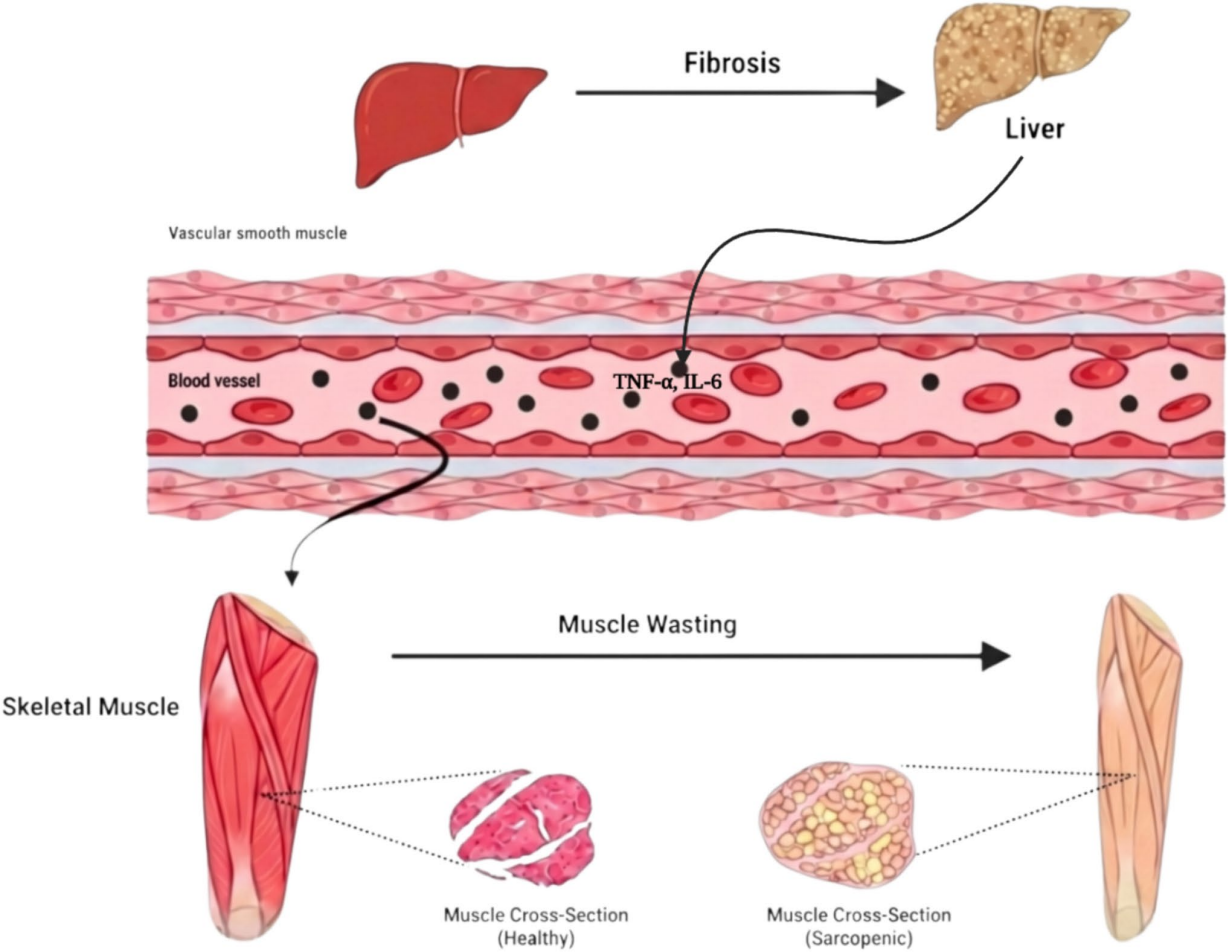
Liver–muscle connection mediated by inflammatory signaling. Liver fibrosis increases the release of the pro-inflammatory mediators TNF-α and IL-6, which enter circulation and contribute to vascular inflammation and downstream skeletal muscle wasting. Elevated TNF-α promotes muscle fiber atrophy, resulting in the transition from healthy muscle cross-sections to sarcopenic morphology

Chronic inflammation is characterized by elevated levels of TNF-α, IL-6, and C-reactive protein (CRP). TNF-α induces oxidative stress and mitochondrial dysfunction, contributing to FLD by inhibiting the AMP-activated protein kinase (AMPK) pathway. IL-6 drives liver inflammation and fibrosis. These cytokines also correlate with reduced muscle mass and strength (Kim and Choi 2019) (Fig. 1). The liver–spleen axis further contributes, with the spleen producing cytokines (TNF-α, IL-6) and immune cells that exacerbate MASLD and sarcopenia (Tarantino et al. 2021).

## Physical inactivity and nutritional factors

Physical inactivity and poor diet exacerbate muscle atrophy and hepatic fat accumulation, impairing protein synthesis and promoting obesity (Kim and Choi 2019). These factors perpetuate a cycle of chronic inflammation, IR, and oxidative stress, worsening both sarcopenia and MASLD (Iwaki et al. 2023).

## Gut microbiota: implications for liver fibrosis and muscle atrophy

The gut microbiota significantly influences liver and muscle health. Dysbiosis—an imbalance in gut microbiota—compromises the intestinal barrier, releasing endotoxins like lipopolysaccharide (LPS) into the bloodstream, triggering systemic inflammation and metabolic disorders that worsen liver fibrosis, steatosis, and other features of MASLD. In the liver, LPS binds to Toll-like receptor 4 (TLR4) on Kupffer cells and hepatic stellate cells (HSCs), activating NF-κB and inducing pro-inflammatory cytokines (e.g., IL-6 and TNF-α) release (Vakilpour et al. 2024). These cytokines, in turn, promote hepatic fibrogenesis and muscle protein breakdown and contribute to sarcopenia (Shen et al. 2017; Tripathi et al. 2018). Consequently, the gut–liver–muscle axis emerges as a vital pathway linking gastrointestinal function to systemic metabolism and regulation.

Tumor necrosis factor alpha (TNF-α) inhibits mTOR signaling in muscle, reducing protein synthesis and increasing degradation via ubiquitin ligases like muscle ring-finger protein 1 (MuRF1) and Atrogin-1 (Keivanlou et al. 2024), while IL-6 exacerbates insulin resistance, further driving muscle wasting (Ponziani et al. 2021). This sustained inflammation not only damages the liver but also perpetuates muscle depletion, establishing a vicious cycle that hastens disease progression. Emerging biomarkers, such as serum zonulin, a marker of intestinal permeability, correlate with sarcopenia severity in cirrhosis patients, with levels above 3.2 ng/mL predicting muscle loss (Ohtani and Kawada 2019). A 2023 longitudinal study revealed that zonulin levels exceeding 3.2 ng/mL predict a 2.3-fold increased risk of progressive muscle loss over one year, independent of liver disease severity (Amini-Salehi et al. 2024).

Therapeutic strategies, such as probiotics (e.g., *Lactobacillus strains*) and prebiotics (e.g., *resistant starch*), restore gut barrier function, reduce inflammation, lower zonulin levels, and preserve muscle mass. In clinical practice, measuring zonulin could help detect high-risk patients earlier, allowing for the timely application of microbiome modulation directed at improving patient outcomes (Keivanlou et al. 2024). Probiotics improve liver function by reducing hepatic inflammation. VSL#3® probiotic therapy enhances liver enzyme profiles and reduces fat accumulation in MASLD patients (Liu et al., 2018). Similarly, *Lactobacillus rhamnosus GG (LGG)* supplementation has been shown to improve liver function in chronic liver disease by restoring microbial homeostasis and reducing systemic inflammation (Derosa et al. 2022).

Probiotics—nondigestible compounds that promote beneficial bacteria—enhance short-chain fatty acid (SCFA) production, like butyrate, which are essential for gut integrity and reducing inflammation, preserving muscle mass and strength (Prokopidis et al. 2023). The combination of prebiotics and probiotics into symbiotics restores microbial balance, reducing gut permeability and improving both hepatic and muscle health (Hadi et al. 2019; Sharpton et al. 2019). Fecal microbiota transplantation (FMT) shows promise in restoring gut health, reducing systemic inflammation, and endotoxemia (Zhang et al. 2023a, b). FMT is still being considered clinically; however, its potential to improve muscle function by mitigating inflammation-driven muscle wasting remains to be seen (De Groot et al. 2017).

## Hepatokines and myokines: mediators of liver–muscle communication

Hepatokines and myokines are critical signaling molecules that mediate liver–muscle communication, influencing various metabolic processes. Their dysregulation contributes to metabolic syndrome, like MASLD, and muscle atrophy (Nishikawa et al. 2024).

Liver secreting hepatokines such as fetuin-A, FGF 21, angiopoietin-related growth factor (ANGPTL6), insulin-like growth factors (IGF), selenoprotein P (SeP), and leukocyte-derived chemotaxin 2 (LECT2), regulate the processes of MASLD and other metabolic co-morbidities (Ke et al. 2019). Fetuin-A promotes insulin resistance and inflammation, thereby contributing to the development of MASLD. Overexpression of fetuin-A activates NF-kB and ERK-1/ERK-2 signaling pathways, thereby stimulating macrophage migration and polarization in adipose tissues. FGF21 enhances insulin sensitivity and reduces oxidative stress, protecting against MASLD. ANGPTL6 modulates glucose metabolism and may influence MASLD progression (Ke et al. 2019).

**Fig. 2:**
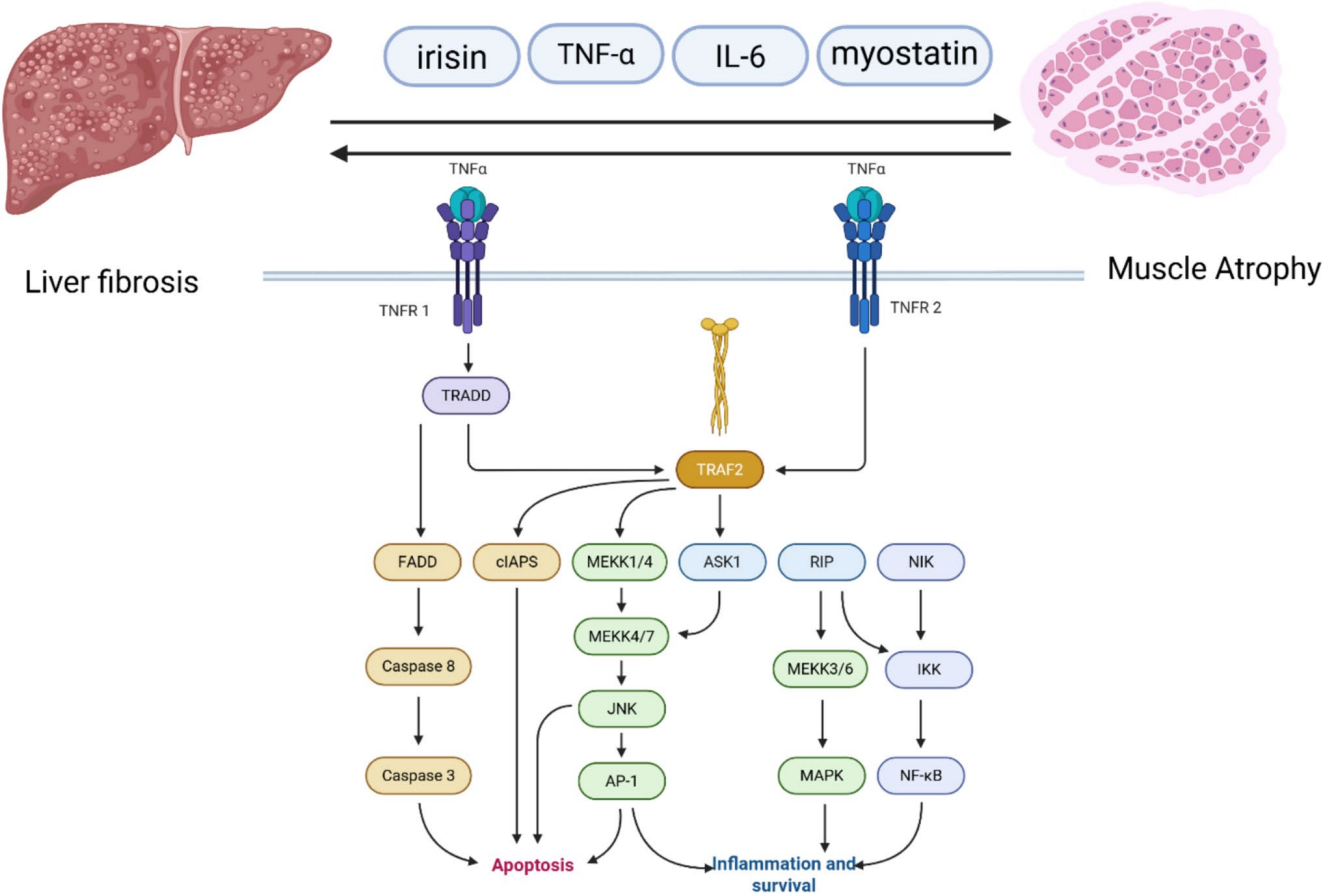
Molecular pathways linking liver fibrosis to skeletal muscle atrophy. Liver fibrosis promotes the release of circulating mediators, including irisin, tumor necrosis factor-alpha (TNF-α), interleukin-6 (IL-6), and myostatin, which interact with TNF receptors (TNFR1 and TNFR2) on skeletal muscle cells. Activation of TNFR1 triggers recruitment of TRADD and FADD, leading to caspase-8 and caspase-3 activation and subsequent apoptosis. In parallel, TNFR1-associated cIAPs and MEKK1/4–MEKK4/7 signaling activate JNK and AP-1, contributing to muscle catabolism. TNFR2 engagement primarily signals through TRAF2, facilitating ASK1, RIP, NIK, and downstream MEKK3/6, MAPK, and NF-κB activation, resulting in inflammation, impaired anabolic signaling, and muscle wasting. Collectively, these pathways illustrate how liver-derived inflammatory and metabolic factors drive molecular events culminating in muscle atrophy. TNF-α, tumor necrosis factor-alpha; IL-6, interleukin-6; TNFR1/2, tumor necrosis factor receptors 1 and 2; TRADD, TNFR1-associated death domain protein; FADD, Fas-associated death domain; cIAPs, cellular inhibitors of apoptosis proteins; TRAF2, TNF receptor–associated factor 2; ASK1, apoptosis signal-regulating kinase 1; RIP, receptor-interacting protein; NIK, NF-κB-inducing kinase; MEKK, MAPK/ERK kinase kinase; JNK, c-Jun N-terminal kinase; AP-1, activator protein-1; MAPK, mitogen-activated protein kinase; NF-κB, nuclear factor kappa-B

Skeletal muscle releases myokines such as irisin, IL-6, and myostatin. Irisin, released after acute bouts of aerobic and resistance exercise, improves insulin sensitivity, promotes weight loss, and reduces hepatic steatosis. IL-6, secreted into the bloodstream in response to physical exertion, facilitates the uptake of glucose by peripheral tissues while concurrently diminishing the deposition of visceral adipose tissue. Myostatin, a myokine that imposes a ceiling on hypertrophic potential, has been associated with impaired insulin sensitivity; engagement in regular training attenuates myostatin concentrations, thereby supporting the maintenance of skeletal muscle mass and metabolic health (Gonzalez-Gil and Elizondo-Montemayor 2020) (Fig. 2).

## Liver–heart crosstalk: comparative insights with skeletal muscle

Emerging data reveal that the liver and the heart engage in a dynamic, bidirectional metabolic and inflammatory dialogue extending beyond classical endocrine signaling. Following myocardial injury or hemodynamic stress, the heart releases IL-6, which activates hepatic STAT3, leading to suppression of hepatocyte mineralocorticoid receptor and induction of fibroblast growth factor-21 (FGF21). The secreted FGF21 then exerts protective effects on cardiomyocytes, mitigating remodeling and preserving function. This IL-6/STAT3–MR–FGF21 axis defines a molecular framework for heart–liver communication after infarction or overload injury (Sun et al. 2007). Conversely, under non-ischemic pressure-overload conditions, hepatic FGF21 secretion drives a feed-forward hepato-cardiac signaling loop that promotes cardiac hypertrophy, suggesting context-dependent effects of the same mediator (Mia et al. 2025). These observations emphasize that the liver does not merely respond to systemic stress but actively modulates cardiac structure and metabolism.

Beyond FGF21, a broader spectrum of hepatokines, including fetuin-A, angiopoietin-like proteins, and ceruloplasmin, contribute to cardiac energy homeostasis and inflammatory tone. FGF21 itself coordinates lipid oxidation, mitochondrial function, autophagy, and oxidative-stress defense in the myocardium, thereby linking hepatic lipid metabolism to cardiovascular outcomes (Harrison et al. 2024). Thus, the liver–heart axis represents an integrated metabolic network that complements the better-known liver–skeletal muscle connection.

## Comparative aspects of cardiac and skeletal muscle signaling

Cardiomyocytes and skeletal myofibers share several regulatory nodes yet differ markedly in their energetic priorities. Both depend on AMP-activated protein kinase (AMPK) and mTORC1 as reciprocal nutrient sensors: AMPK activation during energy stress suppresses mTORC1, thereby maintaining metabolic flexibility (Smiles et al. 2024). However, cardiac tissue is intrinsically more oxidative and relies predominantly on fatty-acid oxidation even under stress, whereas skeletal muscle readily shifts toward glucose utilization. This divergence reflects distinct AMPK isoform compositions, α2/β2/γ1–γ2 complexes predominate in cardiomyocytes, yielding differential responses to energetic and hormonal cues (Fulghum and Hill 2018; Rakoubian et al. 2025). Moreover, inflammatory mediators such as tumor necrosis factor-α (TNF-α) provoke structural remodeling and contractile dysfunction in the heart but mainly induce proteolysis and atrophy in skeletal muscle (Sun et al. 2007; Schumacher and Naga Prasad 2018). These tissue-specific consequences underscore the necessity of distinguishing cardiac from skeletal muscle responses within systemic inflammatory or metabolic stress.

## Clinical implications of liver–heart coupling

At the clinical level, MASLD has emerged as a powerful independent predictor of cardiovascular disease across diverse populations, including younger cohorts (Liao et al. 2024; Zhang et al. 2025). Mechanistically, hepatic lipid overflow and mitochondrial dysfunction may contribute to myocardial energetic impairment and lipotoxic cardiomyopathy (Zhao et al. 2023). In advanced liver disease, cirrhotic cardiomyopathy (CCM), defined by systolic or diastolic dysfunction in the absence of prior heart disease, represents a prototypical manifestation of liver-derived cardiac impairment, warranting echocardiographic surveillance and refined diagnostic criteria (Liu et al. 2024). Collectively, these findings highlight the need for integrated cardiovascular assessment in hepatology practice and vice versa.

Although experimental data substantiate multiple molecular routes of liver–heart communication, direct evidence in humans remains limited, with most mechanistic insights derived from animal or ex vivo studies. Translational investigations employing paired hepatic–cardiac biomarkers and multimodal imaging will be essential to clinically validate these pathways. Furthermore, FGF21’s effects appear context-dependent, cardioprotective after acute infarction yet potentially pro-hypertrophic during chronic pressure overload, underscoring the importance of disease context, timing, and dosing in any therapeutic application (Mia et al. 2025). Future research should also explore multi-omics approaches to map the integrated hepatic-cardiac-skeletal muscle interactome and identify biomarkers predictive of cross-organ metabolic stress.

## Inflammation and oxidative stress: common threads in liver fibrosis and muscle atrophy

Inflammation and oxidative stress act synergistically to unify liver fibrosis and muscle atrophy at the mechanistic level. Elucidation of the pathways common to both syndromes opens avenues for therapies that simultaneously neutralize inflammatory mediators and counteract oxidative damage, thereby enhancing prognostic efficacy. In liver fibrosis, circulating concentrations of TNF-α and IL-6 rise in accordance with disease progression, with muscle cells contributing to the cytokine milieu in addition to the damaged hepatocytes (Steensberg et al. 2002).

Tumor necrosis factor-alpha (TNFα), synthesized in a fibrotic liver, can be released into the circulation and subsequently accumulate in skeletal muscle, where it facilitates muscle wasting by impairing myofibrillar protein synthesis and amplifying proteolytic degradation through the activation of muscle-selective ubiquitin–proteasome components, including MuRF1 (Shirakami et al. 2023). This aberration contributes to the clinical phenotype of cachexia, distinguished by unintentional weight decline and depletion of lean mass (Ji et al. 2022).

Oxidative stress is a key driver of liver fibrosis. Reactive oxygen species (ROS) can activate NF-κB and the NLRP3 (NOD-like receptor protein 3) inflammasome, thereby promoting inflammation and the fibrogenic response in hepatic tissue. In addition, the persistent accumulation of ROS causes direct cellular damage, which accelerates the progression of hepatic fibrosis. Elevated ROS levels similarly impair muscle cells, disrupt mitochondrial function, and diminish the cells’ capacity to produce ATP and preserve structural integrity. This dysfunction facilitates muscle wasting, especially in chronic conditions such as liver fibrosis. The cumulative burden of persistent inflammation and oxidative stress can exceed the endogenous antioxidant capacity of the liver and skeletal muscle, thereby increasing tissue injury. Experimental data indicate that bolstering antioxidant defenses may alleviate specific detrimental manifestations linked to liver fibrosis and concomitant muscle wasting (Ramos-Tovar and Muriel 2020).

## Mitochondrial metabolism and energy crosstalk across the liver–muscle–heart axis

Mitochondrial metabolism plays an integrative role in coordinating energy homeostasis between the trilogy of liver, skeletal muscle, and the heart (Abel 2018). When mitochondrial function becomes impaired in any of these tissues, compensatory and pathological responses propagate across the entire axis, contributing to the clinical presentation of MASLD, sarcopenia, and cardiometabolic dysfunction (Pereyra et al. 2023).

In the liver, impairments in mitochondrial pathways promote hepatic lipid accumulation, ROS generation, and hepatokines’ release. These factors act systemically to modulate muscle insulin sensitivity, mitochondrial biogenesis, and protein turnover (Grossini et al. 2021). Excess hepatic ROS and altered NAD +/NADH ratios further disrupt whole-body energy homeostasis by impairing AMPK signaling and limiting metabolic flexibility in downstream tissues (Nikolic 2024).

Skeletal muscle is similarly dependent on mitochondrial quality control pathways to maintain contractile function and substrate oxidation. Muscle mitochondrial dysfunction reduces fatty acid oxidation, increases intramyocellular lipid intermediates, and activates inflammatory and proteolytic pathways (e.g., NF-κB, FoxO, and MuRF1/Atrogin-1) (Shaikh et al. 2025). The heart adds a layer of metabolic interdependence. The heart, which relies heavily on oxidative phosphorylation, is equally affected by systemic disturbances in lipid handling, ROS levels, and substrate availability. Altered hepatic output or impaired muscle metabolism can disrupt cardiac energy supply, while cardiac mitochondrial stress can influence peripheral metabolism through neurohumoral pathways (Ranjbarvaziri et al. 2021).

Overall, mitochondrial metabolism forms a bidirectional energy-coupling network. Dysfunction in any component of the liver–muscle–heart axis amplifies metabolic stress across the system, contributing to MASLD, sarcopenia, and cardiometabolic complications (Table 1).

**Table 1:** Key molecular mediators of the liver–muscle axis

Mediator	Type	Primary source	Mechanistic actions	Effect on liver	Effect on muscle
FGF21	Hepatokine (also cardiac-induced) (Milani et al. 2024)	Liver, stressed cardiomyocytes (Croon et al. 2022)	Activates AMPK/PPAR pathways; enhances fatty acid oxidation; improves insulin sensitivity (Heinle et al. 2023)	Reduces steatosis, enhances lipid metabolism (Cao et al. 2025)	Improves glucose uptake, enhances mitochondrial oxidative capacity (Sun et al. 2021 )
Fetuin-A or AHSG	Hepatokine	Liver	Promotes insulin resistance; activates TLR4-mediated inflammation (Benomar and Taouis 2019)	Worsens steatosis and hepatic inflammation (Benomar and Taouis 2019)	Impairs insulin signaling and anabolism (Willcockson et al. 2025)
Selenoprotein P (SeP)	Hepatokine	Liver	Antioxidant enzyme; regulates selenium transport; suppresses AMPK (Usui et al. 2023)	Exacerbates insulin resistance and lipid accumulation (Schwarz et al. 2023)	Reduces AMPK activity, promotes muscle fatigue (Usui et al. 2023)
Myostatin or GDF-8	Myokine (negative regulator)	Skeletal muscle	Inhibits myogenesis via Smad2/3; suppresses protein synthesis (AbdelHafez et al. 2022)	Promotes hepatic fibrosis and worsens steatosis (Ruiz-Margáin et al. 2023)	Causes muscle atrophy; reduces strength (Lee 2023)
Irisin	Myokine	Skeletal muscle	Induces browning of adipose tissue; enhances mitochondrial biogenesis (Luo et al. 2022)	Improves steatosis and glucose homeostasis (Yano et al. 2021)	Enhances metabolism and muscle quality (Zhao et al. 2024)
IL-6	Cytokine/myokine	Muscle (exercise-induced), immune cells (Orange et al. 2023)	Acute: activates AMPK (Nash et al. 2023); chronic: activates JAK/STAT inflammatory pathways (Huang et al. 2022 )	Acute improves metabolism; chronic promotes steatosis (Giraldez et al. 2021)	Acute enhances glucose uptake; chronic induces catabolism (Kistner et al.^2022^)
TNF-α	Inflammatory cytokine	Adipose, liver, and immune cells	Activates NF-κB; promotes insulin resistance and proteolysis (Szukiewicz 2023)	Worsens inflammation and fibrosis (Vachliotis and Polyzos 2023)	Promotes muscle wasting and mitochondrial dysfunction (Chen et al. 2023b)
Myonectin or CTRP15	Myokine	Skeletal muscle	Enhances hepatic fatty-acid uptake; regulates lipid homeostasis (Shin 2021)	Reduces circulating lipids; improves hepatic lipid clearance (Chen et al. 2023a)	Indirect metabolic support (Shin 2021)
Adiponectin	Adipokine with muscle–liver influence (de Oliveira Santos et al. 2021)	Adipose tissue	Activates AMPK/PPAR-α; anti-inflammatory (Song et al. 2022)	Improves steatosis, reduces inflammation (Mavilia and Wu 2021)	Enhances fatty-acid oxidation and insulin sensitivity (Lopez-Yus et al. 2022)
SCFAs	Gut-derived metabolites (Cai et al. 2024)	Microbiota fermentation (Cai et al. 2024)	Act on GPR41/43; enhance GLP-1; promote AMPK (Tian et al. 2024)	Improve steatosis, reduce inflammation (Tian et al. 2024)	Enhance muscle insulin sensitivity & mitochondrial biogenesis (Cai et al. 2024)
LPS	Gut-derived endotoxin (Liu et al. 2022)	Gram-negative bacteria	Activates TLR4 → systemic inflammation (Kawaguchi and Torimura 2021)	Drives steatohepatitis (Liu et al. 2022)	Promotes muscle catabolism (Kawaguchi and Torimura 2021)

## Alcoholic liver disease and the liver–muscle axis

Alcoholic liver disease (ALD), including alcoholic steatohepatitis (ASH), represents a clinically meaningful context in which liver–muscle crosstalk is profoundly deregulated, despite not being classically categorized as a metabolic disorder (Dasarathy and Brown 2017). Chronic alcohol consumption has been strongly associated with skeletal muscle wasting and sarcopenia, which in turn negatively affects ALD progression, treatment tolerance, and overall survival in patients. Emerging evidence indicates that this mutual interaction is mediated through several metabolic, inflammatory, and mitochondrial pathways shared with non-alcoholic liver diseases (Simon et al. 2023).

One of the key pathogenic mechanisms linking hepatic injury to muscle dysfunction in ALD is hyperammonemia. Elevated circulating ammonia directly promotes muscle proteolysis and inhibits protein synthesis by activating autophagy and suppressing mTOR signaling in skeletal muscle (Kumar et al. 2017). Skeletal muscle, in turn, acts as an auxiliary site for ammonia detoxification via glutamine synthesis, thereby establishing a compensatory yet maladaptive liver–muscle axis in advanced conditions (Holeček 2022).

Alcohol-induced mitochondrial dysfunction represents another critical contributor. Ethanol metabolism results in overproduction of ROS and acetaldehyde, thereby impairing mitochondrial function in both hepatocytes and myocytes. In skeletal muscle, such alterations contribute to increased mitophagy and diminished contractile function. Such mitochondrial distress is similar to metabolic liver diseases, pointing to shared mechanistic frames across different etiologies (Holeček 2022).

Inflammatory signaling further amplifies liver–muscle communication in ALD. Chronic alcohol exposure promotes systemic inflammation, which drives muscle catabolism and impairs myogenesis (Kumar et al. 2017; Holeček 2022). Conversely, alcohol-related muscle wasting is associated with altered myokine secretion, which may negatively influence hepatic regeneration and metabolic homeostasis. This reciprocal disturbance of hepatokine–myokine signaling reinforces disease severity at both organ levels (de Oliveira Santos et al. 2021).

Importantly, sarcopenia in ALD and ASH is now recognized as an independent predictor of poor clinical outcomes, including increased susceptibility to infections, hepatic decompensation, and mortality. These observations highlight the liver–muscle axis as a clinically relevant therapeutic target in ALD (Dasarathy et al. 2017); Elsabaawy et al. 2025). Interventions aimed at reducing ammonia burden, improving mitochondrial function, and restoring muscle mass may therefore confer dual benefits by alleviating both hepatic and extrahepatic complications.

## Joint therapeutic approaches: integrating diet, exercise, and pharmacological interventions

**Fig. 3:**
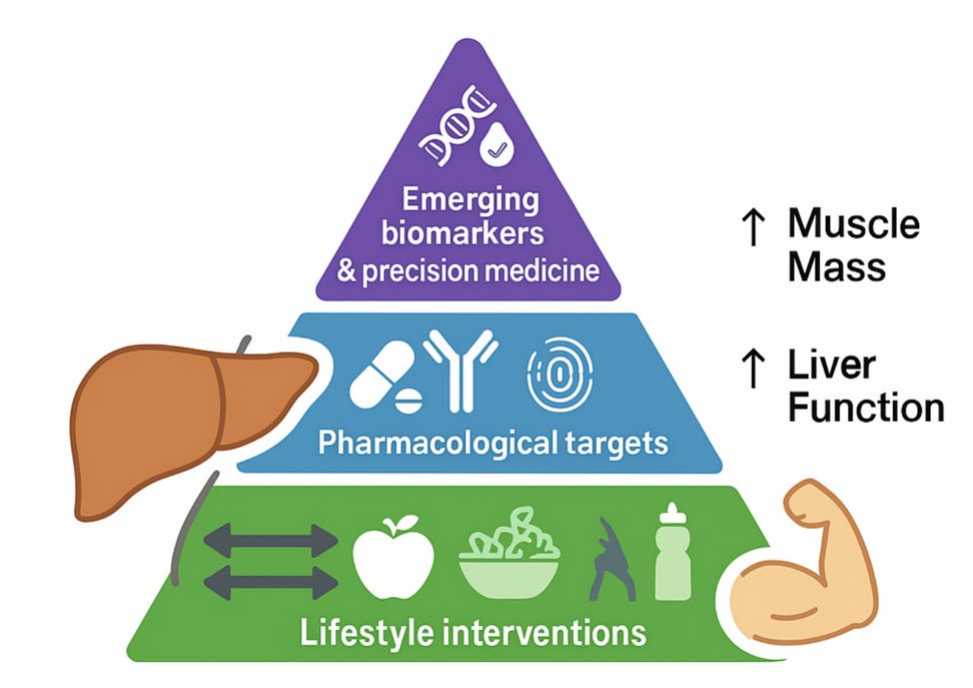
Therapeutic intervention pyramid for liver–muscle health. The pyramid illustrates a tiered approach to improving liver function and muscle mass. Lifestyle interventions form the foundation (nutrition, physical activity, and behavioral changes), followed by pharmacological targets (medications and molecular therapies). At the top, emerging biomarkers and precision medicine represent advanced individualized therapeutic strategies

A joint therapeutic paradigm that integrates dietary, exercise, and pharmacologic strategies provided a robust framework for addressing liver fibrosis and muscle mass loss. Such a paradigm dimension, addressing the intricate metabolic web linking hepatic and skeletal muscle health and highlighting the necessity of comprehensive treatment regimens that engage all dimensions of patient management (Fig. 3).

## Dietary interventions

Dietary interventions involve a well-balanced diet rich in whole foods, including fruits, vegetables, lean meats, and healthy fats. Specific nutritional components, such as antioxidant-rich foods (e.g., berries and nuts), can help reduce inflammation and oxidative stress. Omega-3 fatty acids, abundant in fish oil, help decrease hepatic fat formation and enhance muscle protein synthesis (Soleimani et al. 2019). Additionally, caloric restriction paired with nutrient-dense diets supports weight loss and improves liver function in patients with MASLD or obesity-related conditions. In some instances, weight loss has been shown to reverse liver fibrosis (Vuille-Lessard et al. 2021).

Adequate protein intake is essential for maintaining muscle mass, especially in conditions such as sarcopenia associated with liver disease (Di Cola et al. 2024). The type, quantity, and timing of protein consumption are critical factors. High-quality proteins, such as whey, are more effective than other proteins at promoting muscle protein synthesis due to their rich amino acid profiles (Devries and Phillips 2015). Research demonstrates that whey protein increases muscle protein synthesis by 20–35% more than casein or plant proteins, primarily due to its elevated leucine content (Gorissen et al. 2018). However, excessive consumption of low-quality proteins, such as red and processed meats, can harm liver health (Zhou et al. 2024). A moderate intake of 1.2–1.5 g/kg/day of high-quality protein is recommended to support muscle health without overburdening the liver (Phillips et al. 2016).

Meal timing and frequency also play a significant role in metabolic disorders, particularly liver metabolic disease. Consuming the majority of calories at night is associated with an increased risk of MASLD. Notably, human studies highlight the effectiveness of time-restricted fasting (TRF) in combating obesity and MASLD without altering caloric intake or composition, understanding the importance of meal timing in managing metabolic and liver-related conditions (Carneros et al. 2020).

## Pharmacological interventions

Pharmacological interventions, such as Etanercept (a TNFα inhibitor), have the potential to treat muscle atrophy associated with liver fibrosis by targeting the inflammatory pathways that drive muscle breakdown. Inhibiting TNFα signaling may prevent muscle atrophy without affecting the progression of hepatic fibrosis. Early preclinical and clinical studies confirm TNF-α’s role in muscle wasting linked to liver fibrosis. In a mouse model of liver injury induced by bile duct ligation (BDL), elevated circulating TNF-α triggered skeletal muscle wasting via NF-κB activation, upregulating muscle-specific ubiquitin ligases (MuRF1/Atrogin-1) and inhibiting insulin-like growth factor 1 (IGF-1) signaling. Blocking TNF-α with TNFRII-Fc reduced muscle wasting by 40%, highlighting its therapeutic value (Kurosawa et al. 2021).

However, Etanercept (ETN) has consistently demonstrated clinical efficacy. A retrospective cohort study in *BMJ Open Gastroenterology* (n = 1,450) found no significant effect of anti-TNFα medications on cirrhosis or MASLD progression in patients with autoimmune diseases. However, muscle wasting was not evaluated (Tang et al. 2020). Evidence from rheumatoid arthritis studies indicating a beneficial effect on muscle mass is still inconclusive, and more research is needed (Sokolove et al. 2010). An ongoing phase II randomized controlled trial (NCT04520217) is assessing the effects of Etanercept on sarcopenia in cirrhotic patients, with the results still pending (Allen et al. 2021).

Pharmacological agents that boost antioxidant capacity, such as vitamin E or other ROS scavengers, can reduce oxidative stress in liver and muscles. Hepatoprotective agents, meanwhile, target liver inflammation and fibrosis, indirectly supporting muscle health by improving overall metabolic status. For example, drugs that inhibit hepatic stellate cell activation or extracellular matrix synthesis may facilitate liver healing (Chen et al. 2019).

Recent research has focused on ammonia-lowering therapy for hyperammonemia, a common issue in patients with cirrhosis due to hepatic dysfunction, portosystemic shunting, and decreased ureagenesis, which elevates ammonia levels in skeletal muscle. Ammonia-lowering strategies show promise in treating sarcopenia and hepatic endotoxemia in chronic liver disease (CLD). In vivo studies with portacaval anastomosis (PCA) rats demonstrated that ammonia-lowering medications reduced myostatin expression and autophagy markers, while lean body mass, grip strength, and anabolic signaling increased (Allen et al. 2021).

Hormone replacement therapy, particularly testosterone therapy, offers additional therapeutic strategies. Male cirrhosis often exhibits low serum testosterone levels, which decline as the disease progresses. Testosterone therapy improves body composition by reducing fat mass and increasing handgrip strength (HGS). Higher testosterone levels enhance androgen receptor expression, promote cell proliferation, and increase Akt signaling and IGF-1 levels, thereby fostering muscle growth through the activation and replication of stem cells. It also reduces skeletal muscle myostatin levels, inhibiting JNK activation and apoptosis. However, testosterone therapy in cirrhosis patients is complex, with long-term effects still unclear. Thus, evidence supporting its safe and effective use as an adjunctive therapy remains limited (Allen et al. 2021).

## Ketogenic diet and intermittent fasting: potential role in liver fibrosis and muscle preservation

The ketogenic diet (KD) and intermittent fasting (IF) have gained significant attention for their potential therapeutic benefits in managing liver fibrosis, particularly in the context of MASLD, and in preserving muscle mass.

### Ketogenic diet

KD is characterized by a high-fat, very low-carbohydrate diet, typically providing more than 1000 kcal per day. This distinguishes it from more restrictive very low-calorie diets that drastically limit energy intake (Kirkpatrick et al. 2019). By severely reducing carbohydrate consumption, KD lowers blood glucose and insulin levels, which in turn decreases hepatic de novo lipogenesis—a primary contributor to MASLD development (Crosby et al. 2021). Additionally, the state of nutritional ketosis induced by KD leads to the production of ketone bodies, which may suppress appetite, reduce overall energy intake, and promote weight loss. This weight loss is particularly critical for managing moderate MASLD cases (Gibson et al. 2015).

In MASLD, elevated hepatic triglyceride levels arise from three primary sources: dietary free fatty acids, adipose tissue lipolysis, and de novo lipogenesis, predominantly fueled by glucose. Ketone bodies provide additional benefits by mitigating oxidative stress and inflammation associated with obesity, potentially altering the pathophysiology of MASLD. Research demonstrates that ketone bodies reduce the synthesis of NLRP3 inflammasome-mediated interleukins (IL-1β and IL-18) in human monocytes and attenuate IL-1β release in mouse models (Youm et al. 2015).

Despite these advantages, caution is advised when applying KD to advanced liver diseases, such as cirrhosis. In later stages, impaired liver function may disrupt hepatic fat and ketone body metabolism, potentially worsening hepatic stress and nutritional deficiencies (Allen et al. 2021). While evidence suggests KD can reduce liver fat and inflammation in early-stage MASLD, its safety and efficacy in severe liver disease remain uncertain. Clinicians must carefully assess risks versus benefits and tailor treatments to individual patients until more robust clinical data emerge (Wallace et al. 2021).

Beyond liver health, long-term KD shows promise in counteracting sarcopenia, an age-related condition marked by reduced muscle strength and metabolic decline. Positive effects include a shift in muscle fiber types (from type IIb to type IIa), increased markers of neuromuscular junction remodeling, enhanced mitochondrial biogenesis, improved oxidative metabolism and antioxidant capacity, and reduced endoplasmic reticulum stress, protein synthesis, and proteasome activity (Li et al. 2024).

### Intermittent fasting

IF is a dietary approach that promotes weight loss by limiting energy intake to specific time windows. By lowering insulin levels, IF enables the body to use mobilized stored fat for energy. Hunger-induced autophagy facilitates the clearance of damaged cells. Elevated growth hormone during fasting further supports fat loss and muscle preservation. IF can be implemented in various forms, including alternate-day fasting, modified fasting, and time-restricted eating (Soykurt and Tekdemir 2024).

IF has demonstrated potential in obesity management, with studies spanning 14 to 48 weeks showing reductions in weight gain, visceral adiposity, and cardiometabolic risk, as well as improvements in metabolism, muscle function, and glucose homeostasis. It enhances insulin sensitivity, lowers insulin levels, and restores fasting and postprandial glucose levels. Additionally, some research indicates that IF reduces inflammatory cytokines such as TNF-α, IL-1β, and IL-6 concurrently with improvements in glucose balance, further supporting weight loss and metabolic health (Mulas et al. 2023).

## Metabolic syndrome and sarcopenia: converging pathologies in the liver–muscle axis

Metabolic syndrome (Met-S) and sarcopenia are increasingly recognized as interconnected disorders that adversely affect health, particularly through their impact on the liver–muscle axis. These conditions share standard pathophysiological mechanisms, including insulin resistance, chronic inflammation, and metabolic alterations in both muscle and liver tissues, which drive their interrelated progression (Kumar et al. 2022).

## Shared metabolic disruptions

Intricate metabolic disturbances characterize Met-S and sarcopenia. The link between Met-S and sarcopenia stands out as a critical risk factor for the development of sarcopenia (Gluvic et al. 2017; Saklayen 2018). In this state, glycogenesis is inadequately suppressed, protein breakdown is heightened, and protein synthesis is diminished due to compensatory hyperinsulinemia (Bonaldo and Sandri 2013). This hyperinsulinemia also elevates myostatin levels, a protein that inhibits skeletal muscle growth, further reducing muscle mass.

Additionally, IR triggers lipolysis, increasing the release of free fatty acids (FFAs) from adipose tissue, while suppressing the growth hormone (GH)-insulin-like growth factor 1 (IGF1) axis, which is essential for stimulating protein synthesis in skeletal muscle (Kalyani et al. 2014).

Myofibers, particularly type IIb, secrete proteins and myokines that may counteract metabolic abnormalities. However, age-related or disease-induced skeletal muscle loss exacerbates impairments in glucose metabolism. IR also promotes triglyceride accumulation in both liver and skeletal muscle by enhancing glycogenesis, activating sterol regulatory element-binding protein 1c, inhibiting β-oxidation, increasing FFA availability, and altering triglyceride transport (Postic and Girard 2008). Moreover, low lean body mass is associated with an elevated risk of type 2 diabetes mellitus (T2DM) and insulin resistance, with estimates suggesting that up to 15% of T2DM patients with type 2 diabetes also have sarcopenia (Kim et al. 2014).

## Myosteatosis and its role

In contrast, myosteatosis—the infiltration of fat into skeletal muscle—emerges as an early contributor to MASH before sarcopenia develops. This fat deposition heightens insulin resistance, fosters oxidative stress, and initiates a proinflammatory cascade, which impairs insulin signaling and contributes to muscle atrophy (Wong and Yuan 2024).

## Steatotic liver disease and muscle decline

MASLD further compounds these issues, leading to sarcopenia, muscle deterioration, and chronic inflammation (Iwaki et al. 2023). Inflammatory cytokines released by adipose tissue macrophages are thought to accelerate skeletal muscle breakdown (Hong and Choi 2020). Both reduced muscle mass and diminished muscle strength are linked to an increased risk of MASLD, with muscle strength appearing to be more profoundly affected than muscle mass (Roh et al. 2022). A large-scale study involving 52,815 individuals revealed that those with MASLD experienced a more rapid loss of skeletal muscle mass compared to those without the condition (Sinn et al. 2022).

## Liver transplantation and muscle rehabilitation: challenges and opportunities

Liver transplantation and muscle rehabilitation present significant challenges yet also offer promising opportunities for enhancing patient outcomes. Effectively managing the complexities of liver transplantation while optimizing muscle rehabilitation strategies can substantially improve recovery and quality of life for transplant recipients.

Liver transplantation serves as the definitive treatment for end-stage chronic liver disease, yet it carries distinct implications for muscle health. After surgery, patients frequently develop sarcopenia due to prolonged immobility and the impact of immunosuppressive therapies (Leunis et al. 2023). These factors can delay recovery and elevate morbidity. While transplantation restores liver function, supporting muscle recovery through improved metabolism, immunosuppressive agents, such as calcineurin inhibitors, impair protein synthesis and increase myostatin expression, thereby exacerbating muscle loss (Allen et al. 2021). Consequently, targeted muscle rehabilitation—encompassing interventions such as resistance training and tailored nutritional support—becomes crucial for optimizing patient outcomes.

Emerging strategies, such as stem cell therapies, offer potential to enhance graft function and overall metabolism, which may indirectly benefit muscle health (Rashidi et al. 2018). Nevertheless, additional research is essential to develop specialized rehabilitation protocols that address the unique interplay between muscle function and liver recovery in transplant patients.

## Future directions in liver–muscle axis research: unveiling new therapeutic strategies

Emerging therapies targeting the liver–muscle axis show promise for treating metabolic disorders such as MASLD and MASH. These interventions aim to slow disease progression by addressing underlying metabolic dysfunctions. Below are several promising research directions based on recent findings:

## Understanding interorgan communication

- **Lipid signaling pathways:** Emerging findings indicate that specific lipid mediators synthesized by the liver exert a potent influence on skeletal muscle, enhancing the rate at which fatty acids are oxidized. This dialogue is mediated by nuclear receptor families, including liver- and muscle-expressed peroxisome proliferator-activated receptors (PPAR) isoforms, opening the prospect that selective manipulation of these signaling circuits may improve overall metabolic resilience and reduce the incidence of obesity and metabolic diabetes (Gross et al. 2017).
- **TNFα signaling:** Hepatic fibrosis correlates with excessive secretion of TNFα, a cytokine implicated in skeletal muscle wasting. Administration of the soluble TNF receptor Etanercept (TNFRII-Fc) mitigates muscle loss in liver-fibrotic rodent models, underscoring the potential of anti-inflammatory agents to simultaneously attenuate liver scars and preserve muscle. Future efforts should direct randomized, placebo-controlled trials to assess the impact of TNFα blockade on muscle mass and strength outcomes, and fibrosis markers such as serum PRO-C3 across the spectrum of MASLD to cirrhotic states (Kurosawa et al. 2021).

## Pharmacological innovations

**Nuclear receptor targets**: Investigational therapies are increasingly directed toward hepatic nuclear sensor proteins, principally FXR and the PPAR family, which orchestrate lipid handling, inflammatory tone, and fibrogenic processes. Small molecules and biologics that selectively activate or inhibit these orphan receptors have the potential to remodel liver pathology while simultaneously correcting the skeletal muscle metabolic derangements that compound long-term morbidity in patients with liver disease (Chen et al. 2023a).

## Exercise as a therapeutic strategy

**Exercise-induced myokine release:** Consistent, aerobic-type exercise ramps up the output of myokines—proteins released by contracting muscles—enhancing both liver metabolism and overall energy balance. Targeting exercise routines that maximize myokine elevation could therefore become a stand-alone or adjunct therapy for patients facing the double challenge of liver dysfunction and muscle loss (Chen et al. 2023a).

## Investigating gut–liver–muscle interactions

**Gut microbiota influence:** The gut–liver axis modulates both hepatic and muscle metabolism, mediating effects through inflammatory mediators and the selective uptake of nutrients. Future studies should focus on how tailored pre-, pro-, and synbiotics could reshape gut flora and therefore redraw the signaling maps that connect intestine, liver, and muscle, opening doors for novel, modulatory interventions (Wang et al. 2023).

The liver–skeletal muscle nexus reveals pathways ripe for pharmacologic and lifestyle-directed therapies. By focusing on the small number of signaling molecules that mediate liver–muscle crosstalk, clinicians and investigators could launch interventions that simultaneously stabilize liver health and preserve or restore muscle mass in patients with MASLD and sarcopenia. Ongoing inquiry into these circuits will remain pivotal to translating basic findings into clinically effective strategies that improve outcomes in patients with complex metabolic syndromes.

## Clinical monitoring and management of sarcopenia in liver disease

Sarcopenia commonly complicates liver disease and poses a significant threat, worsening patient well-being and increasing risks both prior to and following liver transplant (Leunis et al. 2023). In this context, chronic liver failure, inadequate nutrition, and pervasive inflammation interact to drive muscle loss, thereby underscoring the value of prompt diagnosis and timely treatment in safeguarding patient prognosis (Altajar and Baffy 2020). This section focuses on the practical management of sarcopenia in liver disease, providing clinicians with clear, pragmatic approaches for measuring muscle loss, monitoring its progression, and implementing evidence-based therapies to address this condition in this vulnerable population.

## Monitoring muscle mass and functionality

Monitoring muscle mass and function is vital to enhancing a patient’s recovery trajectory. Clinicians are encouraged to schedule periodic imaging, either computed tomography (CT) or magnetic resonance imaging (MRI), to obtain accurate muscle measurements. Specifically, the skeletal muscle index measured at the third lumbar vertebra, calculated from CT scans, serves as a widely recognized indicator of sarcopenia among patients facing liver disease (Zhou et al. 2024). If CT or MRI is not practical, dual-energy X-ray absorptiometry (DEXA) presents a gentler alternative for evaluating body composition. Bioelectrical impedance analysis (BIA) provides faster measurements but is less precise, particularly in the presence of fluid shifts. To complete the picture, healthcare teams should routinely include performance evaluations like handgrip strength and the Short Physical Performance Battery (SPPB) in regular clinical care; these tests reliably track a patient’s muscle strength and overall mobility (Phillips et al. 2016; Graham et al. 2021; McLeod 2024).

## Nutritional interventions

Good nutrition plays a central role in managing liver disease and preventing muscle loss. Patients should aim for a caloric intake of 25–35 kcal/kg body weight per day, adjusting the target according to their nutritional status and daily activity level (Plauth et al. 2019). To protect muscle mass, protein intake should be optimized, targeting 1.2 to 1.5 g/kg/day to promote muscle protein synthesis and limit muscle breakdown (Tsien et al. 2015; Kraft et al. 2017). Foods high in leucine—such as dairy, lean meats, seafood, soy, and legumes—are particularly beneficial for preserving muscle (Rondanelli et al. 2021). To offset the effects of overnight fasting and prevent muscle loss, several small meals spaced throughout the day, plus a protein- and carbohydrate-rich snack close to bedtime, are recommended (Plank et al. 2008). Following dietary patterns like the Mediterranean diet, which provide substantial anti-inflammatory and antioxidant benefits, may calm liver inflammation and improve metabolic health (Godos et al. 2017). Including low-glycemic-index carbohydrate sources can further enhance insulin sensitivity and reduce liver fat (Plauth et al. 2019). Attention to micronutrient status is equally critical: vitamin D supplements promote muscle function, and omega-3 fatty acids help lower liver inflammation and fat buildup (Konstantakis et al. 2016; Refaat et al. 2022).

## Physical activity and exercise

Regular physical activity is vital for supporting liver function and maintaining muscle health in patients with liver disease. Aerobic exercises, such as walking, cycling, or swimming, performed for at least 150 min per week, can reduce hepatic fat and enhance insulin sensitivity (Keating et al. 2015; Hashida et al. 2017). High-intensity interval training (HIIT), consisting of brief, intense exercise bursts followed by rest, improves cardiovascular fitness with only 75 min weekly (Hallsworth et al. 2011; Sullivan et al. 2012). Resistance exercises targeting major muscle groups, conducted 2–3 times per week with progressive load increases, are critical for fostering muscle hypertrophy and strength (Gerber et al. 2012). Combining aerobic and resistance training may yield synergistic benefits for liver function and muscle preservation (Oh et al. 2017). Clinicians can propose a phased exercise plan: for weeks 1–4, patients may engage in 30 min of moderate-intensity aerobic exercise five times weekly, paired with resistance training (e.g., squats, lunges, and chest presses) twice weekly, consisting of two sets of 12 repetitions. From weeks 5–8, patients can increase aerobic intensity or duration and add a set or increase the resistance training weight (Alabdul Razzak et al. 2025). Exercise regimens must be tailored to the patient’s disease severity and physical capacity, with medical clearance obtained before initiation to ensure safety.

## Pharmacological strategies

Emerging pharmacological approaches provide additional options for managing sarcopenia. Myostatin inhibitors, though not yet in widespread use, are under investigation for their potential to prevent muscle wasting, and clinicians should monitor advancements in this field (Chen et al. 2019). Hormonal therapies, such as testosterone replacement, may be considered in select patients after a thorough risk–benefit analysis, with diligent monitoring of liver function to minimize adverse effects (Luther et al. 2024). Anti-inflammatory agents, such as TNF-α inhibitors (e.g., Etanercept), can reduce systemic inflammation and muscle wasting, benefiting both liver and muscle health (Nemoto et al. 2011). Antioxidants may alleviate oxidative stress, a significant contributor to liver injury and muscle atrophy (Blas-García and Apostolova 2023). Innovative treatments, such as adipose triglyceride lipase (ATGL) inhibitors, show promise in reducing liver fat, inflammation, and fibrosis while enhancing muscle metabolism (Li et al. 2021).

## Multidisciplinary approach and patient education

A multidisciplinary approach is essential for achieving sustained benefits. A collaborative team, including a hepatologist, dietitian, and physical and occupational therapists, should develop a patient-centered, adaptable treatment plan (Dasarathy 2012). Follow-up visits, conducted every 3 to 6 months, should assess muscle mass, strength, and physical activity levels to enable timely adjustments to the care strategy. Patient education is critical; clinicians must inform patients about sarcopenia, its implications for liver disease, and the importance of adhering to nutritional and exercise recommendations (Holland et al. 2010).

Integrating dietary interventions, exercise, and pharmacological treatments into routine clinical practice, alongside clear patient guidance, empowers healthcare providers to alleviate the burden of sarcopenia. This comprehensive approach can enhance survival and quality of life for patients with liver disease (Tsien et al. 2015). Future research should prioritize cost-effective management strategies and clinician education on the impact of sarcopenia to optimize care for this vulnerable population.

## Efficacy of therapeutic interventions: evidence from clinical trials

Multiple studies have explored exercise, dietary changes, and pharmacological interventions to improve muscle and liver function in patients with MASLD and liver cirrhosis. Recent meta-analyses and RCTs demonstrate that targeted interventions can reduce intrahepatic lipid (IHL) content, improve liver enzyme levels, and enhance muscle strength and insulin sensitivity. For example, (Hallsworth et al. 2011), who reported that resistance exercise resulted in a 27% reduction in IHL and a 20% improvement in handgrip strength, independent of weight loss. Similarly, (Keating et al. 2015), who found that moderate-intensity aerobic exercise significantly reduces liver fat and improves liver enzyme levels (ALT and AST). Additional research shows that nocturnal nutritional supplementation enhances nitrogen balance and reduces muscle catabolism (Plank et al. 2008), while testosterone replacement therapy in men with cirrhosis increases muscle mass by approximately 15% (Sinclair et al. 2016).

Meta-analyses reinforce these findings (Keating et al. 2015), who confirmed that both aerobic and resistance training reduce hepatic fat and improve metabolism. A later meta-analysis by Xiong et al. (2021) noted that aerobic exercise significantly lowered metabolic and liver function markers compared to other exercise types. Furthermore, Zelber-Sagi et al. (2014) and Takahashi et al. (2015) demonstrated that simple resistance exercise programs can enhance liver function in MASLD patients (Table 2).

**Table 2:** Key clinical trials and meta-analyses across various intervention modalities

Intervention modality	Key study	Outcomes	Statistical outcome/effect size
Resistance training	(Javidi et al. 2022)	Reduction in IHL; improvement in handgrip strength	*p* < 0.05
	(Zelber-Sagi et al. 2014)	Significant improvement in liver enzymes and liver fat reduction	SMD reported (significant effect)
	(Takahashi et al. 2015)	Simple resistance exercise reduces liver enzymes and improves hepatic markers	*p* < 0.05
Aerobic training	(Keating et al. 2015)	Reduction in liver fat and improvement in ALT and AST levels	SMD ≈ − 0.65 (95% CI: − 0.89 to − 0.41); *p* < 0.05
	(Cuthbertson et al. 2016)	Improvements in IHL content with moderate aerobic exercise	*p* < 0.05
Nutritional intervention	(Plank et al. 2008)	Enhanced nitrogen balance; reduced muscle catabolism in cirrhotic patients	SMD = 0.45, *p* = 0.02
Pharmacological therapy	(Sinclair et al. 2016)	Testosterone therapy increases muscle mass by ~ 15% in men with cirrhosis	*p* < 0.05

Evidence suggests that targeted exercise—both aerobic and resistance—improves liver and muscle health in patients with MASLD or liver cirrhosis, particularly when sustained for 12 weeks or longer. Aerobic exercise is effective at reducing liver fat and improving liver enzymes, whereas resistance training increases muscle strength and helps regulate lipid levels. Nutritional strategies further enhance metabolic health when implemented alongside physical activity. Nonetheless, the influence of these regimens on insulin sensitivity markers remains heterogeneous; specifically, moderate-intensity continuous aerobic training consistently produces more pronounced reductions in fasting insulin concentrations than resistance training.

Taken together, these observations support integrated treatment regimens that combine structured exercise, personalized dietary interventions, and, when indicated, pharmacotherapy to achieve sustained liver health. The evidence also emphasizes that the duration of the intervention and the iterative coupling of modalities are critical to maximizing therapeutic impact.

The literature reflects the intricate communication between hepatocytes and skeletal muscle that underpins whole-body metabolism. These interactions necessitate precisely calibrated interventions to improve patient outcomes. Insulin resistance serves as a recurrent pathophysiological driver, impairing glycemic control and predisposing muscle to catabolism. Comprehensive lifestyle modifications and selective pharmacological agents antagonize these derangements, restoring metabolic equilibrium. Novel hepatokines, notably FGF21 and ANGPTL6, modulate muscle substrate utilization and enhance mitochondrial fatty acid oxidation. Regular exercise regimens, regardless of type, lower liver transaminases, deplete IHL stores, and preserve lean mass. Alternate dietary paradigms, ranging from ketogenic schemes to timed fasting, ameliorate glycemic excursions, attenuate circulating insulin, and diminish the oxidative and inflammatory milieu that accompanies excess adiposity.

## Conclusion

In summary, growing evidence underscores the crucial role of the liver–muscle axis in regulating systemic metabolism, energy balance, and inflammation across multiple organ systems. Hepatokines and myokines such as FGF21, irisin, and myostatin act as molecular bridges that coordinate glucose and lipid metabolism, mitochondrial function, and protein synthesis between hepatic and muscular tissues. Recent findings further highlight that this network extends to the liver–heart axis, wherein hepatic and cardiac tissues communicate through shared signaling pathways involving IL-6, STAT3, and FGF21. These interactions reveal a complex, context-dependent regulatory system that integrates endocrine and autocrine mechanisms across multiple metabolic organs.

While these insights have advanced understanding of metabolic organ crosstalk, caution is warranted in interpreting their translational implications. Current data, though promising, remain derived mainly from preclinical and mechanistic studies, and causality in humans has yet to be conclusively established. Thus, rather than representing a paradigm shift, these findings provide a refined conceptual framework to guide future investigations into the multi-organ regulation of metabolic homeostasis. Continued interdisciplinary research will be essential to delineate the therapeutic potential of targeting these pathways in MASLD, sarcopenia, and cardiovascular disease.

## Limitations of the study

This review is limited by the heterogeneity and scope of the currently available literature. Most mechanistic evidence on liver–muscle and liver–heart crosstalk drives from animal models or cell culture experiments, which may not fully represent human pathophysiology. Furthermore, significant variation exists among studies in the definition of sarcopenia, MASLD, and cardiometabolic endpoints, limiting direct comparisons and meta-analytic synthesis. Another key constraint is the lack of longitudinal human data directly linking hepatic signaling molecules, such as FGF21 or irisin, to skeletal or cardiac functional outcomes. Additionally, few clinical trials have investigated the simultaneous modulation of hepatic and muscular metabolism as an integrated therapeutic strategy.

Finally, emerging data suggest sex-specific and age-dependent differences in hepatic–muscular signaling, yet most published studies feature predominantly middle-aged male cohorts. These demographic limitations reduce generalizability to broader populations, including women, the elderly, and individuals with comorbidities. Addressing these gaps through large-scale, longitudinal, and multi-omics human studies will be critical to validate preclinical findings and guide future interventions targeting multi-organ metabolic crosstalk, which will be addressed in a future review.

## Data Availability

All data is available within the manuscript.

## Abbreviations

4E-BP1: 4E-binding protein 1
ALT: Alanine aminotransferase
AMPK: AMP-activated protein kinase
ANGPTL6: Angiopoietin-related growth factor 6
AST: Aspartate aminotransferase
ATGL: Adipose triglyceride lipase
BDL: Bile duct ligation
BIA: Bioelectrical impedance analysis
CRP: C-reactive protein
CT: Computed tomography
DEXA: Dual-energy X-ray absorptiometry
DNL: *De novo* Lipogenesis
ECM: Extracellular matrix
FGF21: Fibroblast growth factor 21
FLD: Fatty liver disease
FSTL1: Follistatin-like protein 1
GH: Growth hormone
HIIT: High-intensity interval training
HNF4α: Hepatocyte nuclear factor 4-alpha
HSCs: Hepatic stellate cells
IF: Intermittent fasting
IGF-1: Insulin-like growth factor 1
IHL: Intrahepatic lipid
IL-6: Interleukin-6
IRF4: Interferon regulatory factor 4
KD: Ketogenic diet
LECT2: Leukocyte-derived chemotaxin 2
LPS: Lipopolysaccharide
MASLD: Metabolic dysfunction-associated steatotic liver disease
MASH: Metabolic dysfunction-associated steatohepatitis
Met-S: Metabolic syndrome
MRI: Magnetic resonance imaging
mTOR: Mammalian target of rapamycin
mTORC1: Mammalian target of rapamycin complex 1
MuRF1: Muscle RING-finger protein-1
NAFLD: Non-alcoholic fatty liver disease
NASH: Nonalcoholic steatohepatitis
NF-κB: Nuclear factor kappa B
NLRP3: NOD-like receptor protein 3
PCA: Portacaval anastomosis
PPARs: Peroxisome proliferator-activated receptors
ROS: Reactive oxygen species
S6K1: Ribosomal S6 kinase 1
SCFA: Short-chain fatty acid
SeP: Selenoprotein P
SFA: Saturated fatty acid
SPPB: Short Physical Performance Battery
TFA: Trans fatty acids
TGF-β: Transforming growth factor-beta
TLR4: Toll-like receptor 4
TNFα: Tumor necrosis factor alpha
TRF: Time-restricted fasting
T2DM: Type 2 diabetes mellitus

## References

[CR1] AbdelHafez F, Klausen C, Zhu H, Leung PC (2022) Myostatin increases human trophoblast cell invasion by upregulating N-cadherin via SMAD2/3-SMAD4 signaling. Biol Reprod 106(6):1267–1277 35020826 10.1093/biolre/ioab238

[CR2] Abel ED (2018) Mitochondrial dynamics and metabolic regulation in cardiac and skeletal muscle. Trans Am Clin Climatol Assoc 129:266 30166722 PMC6116613

[CR3] Addissouky TA, Ali MM, Sayed IETE, Wang Y (2024) Emerging advanced approaches for diagnosis and inhibition of liver fibrogenesis. Egypt J Intern Med 36(1):19

[CR4] Alabdul Razzak I, Fares A, Stine JG, Trivedi HD (2025) The role of exercise in steatotic liver diseases: an updated perspective. Liver Int 45(1):e16220 39720849 10.1111/liv.16220 PMC12536350

[CR5] Al-Attar AM, Al-Rethea HA (2017) Chemoprotective effect of omega-3 fatty acids on thioacetamide induced hepatic fibrosis in male rats. Saudi J Biol Sci 24(4):956–965 28490971 10.1016/j.sjbs.2016.01.029 PMC5415165

[CR6] Allen SL, Quinlan JI, Dhaliwal A, Armstrong MJ, Elsharkawy AM, Greig CA, Lord JM, Lavery GG, Breen L (2021) Sarcopenia in chronic liver disease: mechanisms and countermeasures. Am J Physiol Gastrointest Liver Physiol 320(3):G241–G257 33236953 10.1152/ajpgi.00373.2020 PMC8609568

[CR7] Altajar S, Baffy G (2020) Skeletal muscle dysfunction in the development and progression of nonalcoholic fatty liver disease. J Clin Transl Hepatol 8(4):414 33447525 10.14218/JCTH.2020.00065 PMC7782111

[CR8] Amini-Salehi E, Hassanipour S, Keivanlou M-H, Shahdkar M, Orang Goorabzarmakhi M, Vakilpour A, Joukar F, Hashemi M, Sattari N, Javid M (2024) The impact of gut microbiome-targeted therapy on liver enzymes in patients with nonalcoholic fatty liver disease: an umbrella meta-analysis. Nutr Rev 82(6):815–830 37550264 10.1093/nutrit/nuad086

[CR9] Anand AC (2017) Nutrition and muscle in cirrhosis. J Clin Exp Hepatol 7(4):340–357 29234200 10.1016/j.jceh.2017.11.001 PMC5719462

[CR10] Benomar Y, Taouis M (2019) Molecular mechanisms underlying obesity-induced hypothalamic inflammation and insulin resistance: pivotal role of resistin/TLR4 pathways. Front Endocrinol 10:140 10.3389/fendo.2019.00140 PMC6418006 30906281

[CR11] Bertol FS, Araujo B, Jorge BB, Rinaldi N, De Carli LA, Tovo CV (2020) Role of micronutrients in staging of nonalcoholic fatty liver disease: a retrospective cross-sectional study. World J Gastrointest Surg 12(6):269 32774765 10.4240/wjgs.v12.i6.269 PMC7385512

[CR12] Blas-García A, Apostolova N (2023) Novel therapeutic approaches to liver fibrosis based on targeting oxidative stress. Antioxidants 12(8):1567 37627562 10.3390/antiox12081567 PMC10451738

[CR13] Bodine SC, Sinha I, Sweeney HL (2023) Mechanisms of skeletal muscle atrophy and molecular circuitry of stem cell fate in skeletal muscle regeneration and aging. J Gerontol A Biol Sci Med Sci 78(Supplement_1):14–18 37325966 10.1093/gerona/glad023 PMC10272973

[CR14] Bonaldo P, Sandri M (2013) Cellular and molecular mechanisms of muscle atrophy. Dis Model Mech 6(1):25–39 23268536 10.1242/dmm.010389 PMC3529336

[CR15] Cai L, Wang X, Zhu X, Xu Y, Qin W, Ren J, Jiang Q, Yan X (2024) *Lactobacillus*-derived protoporphyrin IX and SCFAs regulate the fiber size via glucose metabolism in the skeletal muscle of chickens. Msystems 9(6):e00214-00224 38780275 10.1128/msystems.00214-24 PMC11237663

[CR16] Cao X, Wang N, Yang M, Zhang C (2025) Lipid accumulation and insulin resistance: bridging metabolic dysfunction-associated fatty liver disease and chronic kidney disease. Int J Mol Sci 26(14):6962 40725208 10.3390/ijms26146962 PMC12295539

[CR17] Carneros D, López-Lluch G, Bustos M (2020) Physiopathology of lifestyle interventions in non-alcoholic fatty liver disease (NAFLD). Nutrients 12(11):3472 33198247 10.3390/nu12113472 PMC7697937

[CR18] Chang K-V, Chen J-D, Wu W-T, Huang K-C, Lin H-Y, Han D-S (2019) Is sarcopenia associated with hepatic encephalopathy in liver cirrhosis? A systematic review and meta-analysis. J Formos Med Assoc 118(4):833–842 30279030 10.1016/j.jfma.2018.09.011

[CR19] Chen Z, Jain A, Liu H, Zhao Z, Cheng K (2019) Targeted drug delivery to hepatic stellate cells for the treatment of liver fibrosis. J Pharmacol Exp Ther 370(3):695–702 30886124 10.1124/jpet.118.256156 PMC6806344

[CR20] Chen C, Xie L, Zhang M, Shama, Cheng KKY, Jia W (2023a) The interplay between the muscle and liver in the regulation of glucolipid metabolism. J Mol Cell Biol 15(12):mjad073 10.1093/jmcb/mjad073 PMC11078061 38095440

[CR21] Chen X, Ji Y, Liu R, Zhu X, Wang K, Yang X, Liu B, Gao Z, Huang Y, Shen Y (2023) Mitochondrial dysfunction: roles in skeletal muscle atrophy. J Transl Med 21(1):503 37495991 10.1186/s12967-023-04369-z PMC10373380

[CR22] Cifuentes M, Verdejo HE, Castro PF, Corvalan AH, Ferreccio C, Quest AF, Kogan MJ, Lavandero S (2025) Low-grade chronic inflammation: a shared mechanism for chronic diseases. Physiology 40(1):4–25 10.1152/physiol.00021.2024 39078396

[CR23] Croon M, Szczepanowska K, Popovic M, Lienkamp C, Senft K, Brandscheid CP, Bock T, Gnatzy-Feik L, Ashurov A, Acton RJ (2022) FGF21 modulates mitochondrial stress response in cardiomyocytes only under mild mitochondrial dysfunction. Sci Adv 8(14):eabn7105 35385313 10.1126/sciadv.abn7105 PMC8986112

[CR24] Crosby L, Davis B, Joshi S, Jardine M, Paul J, Neola M, Barnard ND (2021) Ketogenic diets and chronic disease: weighing the benefits against the risks. Front Nutr 8:702802 34336911 10.3389/fnut.2021.702802 PMC8322232

[CR25] Cui Y, Zhang M, Guo J, Jin J, Wang H, Wang X (2024) Correlation between sarcopenia and cirrhosis: a meta-analysis. Front Nutr 10:1342100 38268669 10.3389/fnut.2023.1342100 PMC10805929

[CR26] Cuthbertson DJ, Shojaee-Moradie F, Sprung VS, Jones H, Pugh CJ, Richardson P, Kemp GJ, Barrett M, Jackson NC, Thomas EL (2016) Dissociation between exercise-induced reduction in liver fat and changes in hepatic and peripheral glucose homoeostasis in obese patients with non-alcoholic fatty liver disease. Clin Sci 130(2):93–104 10.1042/CS20150447 26424731

[CR27] Dasarathy S (2012) Consilience in sarcopenia of cirrhosis. J Cachexia Sarcopenia Muscle 3(4):225–237 22648736 10.1007/s13539-012-0069-3 PMC3505573

[CR28] Dasarathy S, Brown JM (2017) Alcoholic liver disease on the rise: interorgan cross talk driving liver injury. Alcohol Clin Exp Res 41(5):880 28295407 10.1111/acer.13370 PMC5405002

[CR29] Dasarathy J, McCullough AJ, Dasarathy S (2017) Sarcopenia in alcoholic liver disease: clinical and molecular advances. Alcohol Clin Exp Res 41(8):1419–1431 28557005 10.1111/acer.13425 PMC5553706

[CR30] De Bandt J-P, Jegatheesan P, Tennoune-El-Hafaia N (2018) Muscle loss in chronic liver diseases: the example of nonalcoholic liver disease. Nutrients 10(9):1195 30200408 10.3390/nu10091195 PMC6165394

[CR31] De Chiara F, Ureta Checcllo C, Ramon Azcon J (2019) High protein diet and metabolic plasticity in non-alcoholic fatty liver disease: myths and truths. Nutrients 11(12):2985 31817648 10.3390/nu11122985 PMC6950466

[CR32] De Groot PF, Frissen M, De Clercq N, Nieuwdorp M (2017) Fecal microbiota transplantation in metabolic syndrome: history, present and future. Gut Microbes 8(3):253–267 28609252 10.1080/19490976.2017.1293224 PMC5479392

[CR33] de Oliveira Santos AR, de Oliveira Zanuso B, Miola VFB, Barbalho SM, Santos Bueno PC, Flato UAP, Detregiachi CRP, Buchaim DV, Buchaim RL, Tofano RJ (2021) Adipokines, myokines, and hepatokines: crosstalk and metabolic repercussions. Int J Mol Sci 22(5):2639 10.3390/ijms22052639 PMC7961600 33807959

[CR34] Derosa G, Guasti L, D’Angelo A, Martinotti C, Valentino MC, Di Matteo S, Bruno GM, Maresca AM, Gaudio GV, Maffioli P (2022) Probiotic therapy with VSL# 3® in patients with NAFLD: a randomized clinical trial. Front Nutr 9:846873 35685888 10.3389/fnut.2022.846873 PMC9172906

[CR35] Devries MC, Phillips SM (2015) Supplemental protein in support of muscle mass and health: advantage whey. J Food Sci 80(S1):A8–A15 25757896 10.1111/1750-3841.12802

[CR36] Di Cola S, Khan S, Lapenna L, Merli M (2024) Emerging drugs for the treatment of sarcopenia in cirrhosis of the liver. Expert Opin Emerg Drugs 29(2):81–91 38549232 10.1080/14728214.2024.2332428

[CR37] Elsabaawy M, Badran H, Ragab A, Abdelhafiz R, Nageeb M, Ashour R (2025) ALBI-sarcopenia score as a predictor of treatment outcomes in hepatocellular carcinoma. Sci Rep 15(1):14621 40287454 10.1038/s41598-025-97295-7 PMC12033259

[CR38] Fry CS, Drummond MJ, Glynn EL, Dickinson JM, Gundermann DM, Timmerman KL, Walker DK, Dhanani S, Volpi E, Rasmussen BB (2011) Aging impairs contraction-induced human skeletal muscle mTORC1 signaling and protein synthesis. Skelet Muscle 1(1):11 21798089 10.1186/2044-5040-1-11 PMC3156634

[CR39] Fulghum K, Hill BG (2018) Metabolic mechanisms of exercise-induced cardiac remodeling. Front Cardiovasc Med 5:127 30255026 10.3389/fcvm.2018.00127 PMC6141631

[CR40] Gerber L, Otgonsuren M, Mishra A, Escheik C, Birerdinc A, Stepanova M, Younossi Z (2012) Non-alcoholic fatty liver disease (NAFLD) is associated with low level of physical activity: a population-based study. Aliment Pharmacol Ther 36(8):772–781 22958053 10.1111/apt.12038

[CR41] Gibson AA, Seimon RV, Lee CM, Ayre J, Franklin J, Markovic T, Caterson I, Sainsbury A (2015) Do ketogenic diets really suppress appetite? A systematic review and meta‐analysis. Obes Rev 16(1):64–76 25402637 10.1111/obr.12230

[CR42] Giraldez MD, Carneros D, Garbers C, Rose-John S, Bustos M (2021) New insights into IL-6 family cytokines in metabolism, hepatology and gastroenterology. Nat Rev Gastroenterol Hepatol 18(11):787–803 34211157 10.1038/s41575-021-00473-x

[CR43] Giri S, Anirvan P, Angadi S, Singh A, Lavekar A (2024) Prevalence and outcome of sarcopenia in non-alcoholic fatty liver disease. World J Gastrointest Pathophysiol 15(1):91–100 10.4291/wjgp.v15.i1.91100 PMC11045355 38682026

[CR44] Gluvic Z, Zaric B, Resanovic I, Obradovic M, Mitrovic A, Radak D, Isenovic ER (2017) Link between metabolic syndrome and insulin resistance. Curr Vasc Pharmacol 15(1):30–39 27748199 10.2174/1570161114666161007164510

[CR45] Godos J, Federico A, Dallio M, Scazzina F (2017) Mediterranean diet and nonalcoholic fatty liver disease: molecular mechanisms of protection. Int J Food Sci Nutr 68(1):18–27 27484357 10.1080/09637486.2016.1214239

[CR46] Gonzalez-Gil AM, Elizondo-Montemayor L (2020) The role of exercise in the interplay between myokines, hepatokines, osteokines, adipokines, and modulation of inflammation for energy substrate redistribution and fat mass loss: a review. Nutrients 12(6):1899 32604889 10.3390/nu12061899 PMC7353393

[CR47] Gorissen SH, Crombag JJ, Senden JM, Waterval WH, Bierau J, Verdijk LB, Van Loon LJ (2018) Protein content and amino acid composition of commercially available plant-based protein isolates. Amino Acids 50(12):1685–1695 30167963 10.1007/s00726-018-2640-5 PMC6245118

[CR48] Graham ZA, Lavin KM, O’Bryan SM, Thalacker-Mercer AE, Buford TW, Ford KM, Broderick TJ, Bamman MM (2021) Mechanisms of exercise as a preventative measure to muscle wasting. Am J Physiol Cell Physiol 321(7):C40–C57 33950699 10.1152/ajpcell.00056.2021 PMC8424676

[CR49] Gross B, Pawlak M, Lefebvre P, Staels B (2017) PPARs in obesity-induced T2DM, dyslipidaemia and NAFLD. Nat Rev Endocrinol 13(1):36–49 27636730 10.1038/nrendo.2016.135

[CR50] Grossini E, Garhwal DP, Calamita G, Romito R, Rigamonti C, Minisini R, Smirne C, Surico D, Bellan M, Pirisi M (2021) Exposure to plasma from non-alcoholic fatty liver disease patients affects hepatocyte viability, generates mitochondrial dysfunction, and modulates pathways involved in fat accumulation and inflammation. Front Med 8:693997 10.3389/fmed.2021.693997 PMC8282995 34277668

[CR51] Guo S, Feng Y, Zhu X, Zhang X, Wang H, Wang R, Zhang Q, Li Y, Ren Y, Gao X (2023) Metabolic crosstalk between skeletal muscle cells and liver through IRF4-FSTL1 in nonalcoholic steatohepatitis. Nat Commun 14(1):6047 37770480 10.1038/s41467-023-41832-3 PMC10539336

[CR52] Guveli H, Kenger EB, Ozlu T, Kaya E, Yilmaz Y (2021) Macro-and micronutrients in metabolic (dysfunction) associated fatty liver disease: association between advanced fibrosis and high dietary intake of cholesterol/saturated fatty acids. Eur J Gastroenterol Hepatol 33(1S):e390–e394 33731597 10.1097/MEG.0000000000002110

[CR53] Hadi A, Mohammadi H, Miraghajani M, Ghaedi E (2019) Efficacy of synbiotic supplementation in patients with nonalcoholic fatty liver disease: a systematic review and meta-analysis of clinical trials: synbiotic supplementation and NAFLD. Crit Rev Food Sci Nutr 59(15):2494–2505 29584449 10.1080/10408398.2018.1458021

[CR54] Hallsworth K, Fattakhova G, Hollingsworth KG, Thoma C, Moore S, Taylor R, Day CP, Trenell MI (2011) Resistance exercise reduces liver fat and its mediators in non-alcoholic fatty liver disease independent of weight loss. Gut 60(9):1278–1283 21708823 10.1136/gut.2011.242073 PMC3152868

[CR55] Harrison SA, Rolph T, Knott M, Dubourg J (2024) FGF21 agonists: an emerging therapeutic for metabolic dysfunction-associated steatohepatitis and beyond. J Hepatol 81(3):562–576 38710230 10.1016/j.jhep.2024.04.034

[CR56] Hashida R, Kawaguchi T, Bekki M, Omoto M, Matsuse H, Nago T, Takano Y, Ueno T, Koga H, George J (2017) Aerobic vs. resistance exercise in non-alcoholic fatty liver disease: a systematic review. J Hepatol 66(1):142–152 27639843 10.1016/j.jhep.2016.08.023

[CR57] Heinle JW, DiJoseph K, Sabag A, Oh S, Kimball SR, Keating S, Stine JG (2023) Exercise is medicine for nonalcoholic fatty liver disease: exploration of putative mechanisms. Nutrients 15(11):2452 37299416 10.3390/nu15112452 PMC10255270

[CR58] Henin G, Lanthier N, Dahlqvist G (2022) Pathophysiological changes of the liver-muscle axis in end-stage liver disease: what is the right target? Acta Gastroenterol Belg 85:611–624 36566371 10.51821/85.4.10899

[CR59] Holeček M (2022) Muscle amino acid and adenine nucleotide metabolism during exercise and in liver cirrhosis: speculations on how to reduce the harmful effects of ammonia. Metabolites 12(10):971 36295872 10.3390/metabo12100971 PMC9611132

[CR60] Holland AE, Hill CJ, Rasekaba T, Lee A, Naughton MT, McDonald CF (2010) Updating the minimal important difference for six-minute walk distance in patients with chronic obstructive pulmonary disease. Arch Phys Med Rehabil 91(2):221–225 20159125 10.1016/j.apmr.2009.10.017

[CR61] Hong S-h, Choi KM (2020) Sarcopenic obesity, insulin resistance, and their implications in cardiovascular and metabolic consequences. Int J Mol Sci 21(2):494 31941015 10.3390/ijms21020494 PMC7013734

[CR62] Huang B, Lang X, Li X (2022) The role of IL-6/JAK2/STAT3 signaling pathway in cancers. Front Oncol 12:1023177 36591515 10.3389/fonc.2022.1023177 PMC9800921

[CR63] Iwaki M, Kobayashi T, Nogami A, Saito S, Nakajima A, Yoneda M (2023) Impact of sarcopenia on non-alcoholic fatty liver disease. Nutrients 15(4):891 36839249 10.3390/nu15040891 PMC9965462

[CR64] Javidi M, Ahmadizad S, Argani H, Najafi A, Ebrahim K, Salehi N, Javidi Y, Pescatello LS, Jowhari A, Hackett DA (2022) Effect of lower-versus higher-intensity isometric handgrip training in adults with hypertension: a randomized controlled trial. J Cardiovasc Dev Dis 9(9):287 36135432 10.3390/jcdd9090287 PMC9500826

[CR65] Ji Y, Li M, Chang M, Liu R, Qiu J, Wang K, Deng C, Shen Y, Zhu J, Wang W (2022) Inflammation: roles in skeletal muscle atrophy. Antioxidants 11(9):1686 36139760 10.3390/antiox11091686 PMC9495679

[CR66] Jia F, Hu X, Kimura T, Tanaka N (2021) Impact of dietary fat on the progression of liver fibrosis: lessons from animal and cell studies. Int J Mol Sci 22(19):10303 34638640 10.3390/ijms221910303 PMC8508674

[CR67] Jiang S, Kim TM, Park SY, Jin E-J (2025) ROS-responsive MnO2 mesoporous hydrogel to modulate liver-muscle crosstalk and mitigate NAFLD-associated sarcopenia via exosomal miR-582-5p delivery. Theranostics 15(10):4579 40225561 10.7150/thno.108280 PMC11984410

[CR68] Kalyani RR, Corriere M, Ferrucci L (2014) Age-related and disease-related muscle loss: the effect of diabetes, obesity, and other diseases. Lancet Diabetes Endocrinol 2(10):819–829 24731660 10.1016/S2213-8587(14)70034-8 PMC4156923

[CR69] Kawaguchi T, Torimura T (2021) Leaky gut-derived tumor necrosis factor-α causes sarcopenia in patients with liver cirrhosis. Clin Mol Hepatol 28(2):177 34433256 10.3350/cmh.2021.0246 PMC9013621

[CR70] Ke Y, Xu C, Lin J, Li Y (2019) Role of hepatokines in non-alcoholic fatty liver disease. J Transl Intern Med 7(4):143–148 10.2478/jtim-2019-0029 PMC6985917 32010600

[CR71] Keating SE, Hackett DA, Parker HM, O’Connor HT, Gerofi JA, Sainsbury A, Baker MK, Chuter VH, Caterson ID, George J (2015) Effect of aerobic exercise training dose on liver fat and visceral adiposity. J Hepatol 63(1):174–182 25863524 10.1016/j.jhep.2015.02.022

[CR72] Keivanlou M-H, Amini-Salehi E, Sattari N, Hashemi M, Saberian P, Prabhu SV, Javid M, Mirdamadi A, Heidarzad F, Bakhshi A (2024) Gut microbiota interventions in type 2 diabetes mellitus: an umbrella review of glycemic indices. Diabetes Metab Syndr 18(8):103110 39213690 10.1016/j.dsx.2024.103110

[CR73] Kelly RK, Tong TY, Watling CZ, Reynolds A, Piernas C, Schmidt JA, Papier K, Carter JL, Key TJ, Perez-Cornago A (2023) Associations between types and sources of dietary carbohydrates and cardiovascular disease risk: a prospective cohort study of UK Biobank participants. BMC Med 21(1):34 36782209 10.1186/s12916-022-02712-7 PMC9926727

[CR74] Khazaei Y, Dehghanseresht N, Mousavi SE, Nazari M, Salamat S, Asbaghi O, Mansoori A (2023) Association between protein intake from different animal and plant origins and the risk of non-alcoholic fatty liver disease: a case-control study. Clin Nutr Res 12(1):29 36793780 10.7762/cnr.2023.12.1.29 PMC9900076

[CR75] Kim JA, Choi KM (2019) Sarcopenia and fatty liver disease. Hepatol Int 13(6):674–687 31705444 10.1007/s12072-019-09996-7

[CR76] Kim TN, Park MS, Ryu JY, Choi HY, Hong HC, Yoo HJ, Kang HJ, Song W, Park SW, Baik SH (2014) Impact of visceral fat on skeletal muscle mass and vice versa in a prospective cohort study: the Korean Sarcopenic Obesity Study (KSOS). PLoS One 9(12):e115407 25517117 10.1371/journal.pone.0115407 PMC4269440

[CR77] Kirkpatrick CF, Bolick JP, Kris-Etherton PM, Sikand G, Aspry KE, Soffer DE, Willard K-E, Maki KC (2019) Review of current evidence and clinical recommendations on the effects of low-carbohydrate and very-low-carbohydrate (including ketogenic) diets for the management of body weight and other cardiometabolic risk factors: a scientific statement from the National Lipid Association Nutrition and Lifestyle Task Force. J Clin Lipidol 13(5):689-711. e681 31611148 10.1016/j.jacl.2019.08.003

[CR78] Kistner TM, Pedersen BK, Lieberman DE (2022) Interleukin 6 as an energy allocator in muscle tissue. Nat Metab 4(2):170–179 35210610 10.1038/s42255-022-00538-4

[CR79] Konstantakis C, Tselekouni P, Kalafateli M, Triantos C (2016) Vitamin D deficiency in patients with liver cirrhosis. Annals of Gastroenterology: Quarterly Publication of the Hellenic Society of Gastroenterology 29(3):297 10.20524/aog.2016.0037 PMC4923814 27366029

[CR80] Kraft G, Coate KC, Winnick JJ, Dardevet D, Donahue EP, Cherrington AD, Williams PE, Moore MC (2017) Glucagon’s effect on liver protein metabolism in vivo. Am J Physiol Endocrinol Metab 313(3):E263–E272 28536182 10.1152/ajpendo.00045.2017 PMC5625084

[CR81] Kumar A, Davuluri G, Silva RNe, Engelen MP, Have G. A. Ten, Prayson R, Deutz NE, Dasarathy S (2017) Ammonia lowering reverses sarcopenia of cirrhosis by restoring skeletal muscle proteostasis. Hepatology 65(6):2045–2058 28195332 10.1002/hep.29107 PMC5444955

[CR82] Kumar R, Prakash SS, Priyadarshi RN, Anand U (2022) Sarcopenia in chronic liver disease: a metabolic perspective. J Clin Transl Hepatol 10(6):1213 36381104 10.14218/JCTH.2022.00239 PMC9634780

[CR83] Kurosawa T, Goto M, Kaji N, Aikiyo S, Mihara T, Ikemoto-Uezumi M, Toyoda M, Kanazawa N, Nakazawa T, Hori M (2021) Liver fibrosis-induced muscle atrophy is mediated by elevated levels of circulating TNFα. Cell Death Dis 12(1):11 33414474 10.1038/s41419-020-03353-5 PMC7791043

[CR84] Lee S-J (2023) Myostatin: a skeletal muscle chalone. Annu Rev Physiol 85(1):269–291 36266260 10.1146/annurev-physiol-012422-112116 PMC10163667

[CR85] Leunis S, Vandecruys M, Van Craenenbroeck A, Cornelissen V, Bogaerts S, De Smet S, Monbaliu D (2023) Sarcopenia in end-stage liver disease and after liver transplantation. Acta Gastroenterol Belg 86(2):323–334 37428166 10.51821/86.2.11412

[CR86] Li T, Guo W, Zhou Z (2021) Adipose triglyceride lipase in hepatic physiology and pathophysiology. Biomolecules 12(1):57 35053204 10.3390/biom12010057 PMC8773762

[CR87] Li J, Zhang S, Li C, Zhang X, Shan Y, Zhang Z, Bo H, Zhang Y (2024) Endurance exercise-induced histone methylation modification involved in skeletal muscle fiber type transition and mitochondrial biogenesis. Sci Rep 14(1):21154 39256490 10.1038/s41598-024-72088-6 PMC11387812

[CR88] Liao Y-L, Zhu G-Y, Chang C (2024) Non-alcoholic fatty liver disease increases the risk of cardiovascular disease in young adults and children: a systematic review and meta-analysis of cohort studies. Front Cardiovasc Med 10:1291438 38268853 10.3389/fcvm.2023.1291438 PMC10806083

[CR89] Liu Y, McClain C, Feng W (2018) Probiotic *Lactobacillus rhamnosus* GG attenuates BDL‐induced liver injury through reduction of hepatic bile acid accumulation and induction of gut bile acid excretion in mice. FASEB J 32:701.711-701.711

[CR90] Liu Y, Zhang X, Chen S, Wang J, Yu S, Li Y, Xu M, Aboubacar H, Li J, Shan T (2022) Gut-derived lipopolysaccharide promotes alcoholic hepatosteatosis and subsequent hepatocellular carcinoma by stimulating neutrophil extracellular traps through toll-like receptor 4. Clin Mol Hepatol 28(3):522 35508957 10.3350/cmh.2022.0039 PMC9293619

[CR91] Liu H, Naser JA, Lin G, Lee SS (2024) Cardiomyopathy in cirrhosis: from pathophysiology to clinical care. JHEP Rep 6(1):100911 38089549 10.1016/j.jhepr.2023.100911 PMC10711481

[CR92] Lopez-Yus M, Lopez-Perez R, Garcia-Sobreviela MP, Del Moral-Bergos R, Lorente-Cebrian S, Arbones-Mainar JM (2022) Adiponectin overexpression in C2C12 myocytes increases lipid oxidation and myofiber transition. J Physiol Biochem 78(2):517–525 34423393 10.1007/s13105-021-00836-7

[CR93] Luo X, Li J, Zhang H, Wang Y, Shi H, Ge Y, Yu X, Wang H, Dong Y (2022) Irisin promotes the browning of white adipocytes tissue by AMPKα1 signaling pathway. Res Vet Sci 152:270–276 36063604 10.1016/j.rvsc.2022.08.025

[CR94] Luther PM, Spillers NJ, Talbot NC, Sinnathamby ES, Ellison D, Kelkar RA, Ahmadzadeh S, Shekoohi S, Kaye AD (2024) Testosterone replacement therapy: clinical considerations. Expert Opin Pharmacother 25(1):25–35 38229462 10.1080/14656566.2024.2306832

[CR95] Mahadevan V (2020) Anatomy of the liver. Surg Oxf 38(8):427–431

[CR96] Mavilia MG, Wu GY (2021) Liver and serum adiponectin levels in non‐alcoholic fatty liver disease. J Dig Dis 22(4):214–221 33675573 10.1111/1751-2980.12980

[CR97] McLeod KJ (2024) Reversal of soleus muscle atrophy in older adults: a non-volitional exercise intervention for a changing climate. Clin Interv Aging. 10.2147/cia.s447665 38745745 10.2147/CIA.S447665 PMC11093118

[CR98] Mia S, Siokatas G, Sidiropoulou R, Hoffman M, Fragkiadakis K, Markopoulou E, Elesawy MI, Roy R, Blair S, Kuwabara Y (2025) Hepato-cardiac interorgan communication controls cardiac hypertrophy via combined endocrine-autocrine FGF21 signaling. Cell Rep Med. 10.1016/j.xcrm.2025.102125 40339570 10.1016/j.xcrm.2025.102125 PMC12208340

[CR99] Milani I, Codini M, Guarisco G, Chinucci M, Gaita C, Leonetti F, Capoccia D (2024) Hepatokines and MASLD: the GLP1-Ras-FGF21-fetuin-A crosstalk as a therapeutic target. Int J Mol Sci 25(19):10795 39409124 10.3390/ijms251910795 PMC11477334

[CR100] Mukund K, Subramaniam S (2020) Skeletal muscle: a review of molecular structure and function, in health and disease. Wiley Interdiscip Rev Syst Biol Med 12(1):e1462 31407867 10.1002/wsbm.1462 PMC6916202

[CR101] Mulas A, Cienfuegos S, Ezpeleta M, Lin S, Pavlou V, Varady KA (2023) Effect of intermittent fasting on circulating inflammatory markers in obesity: a review of human trials. Front Nutr 10:1146924 37139450 10.3389/fnut.2023.1146924 PMC10149732

[CR102] Nash D, Hughes MG, Butcher L, Aicheler R, Smith P, Cullen T, Webb R (2023) IL‐6 signaling in acute exercise and chronic training: potential consequences for health and athletic performance. Scand J Med Sci Sports 33(1):4–19 36168944 10.1111/sms.14241 PMC10092579

[CR103] Nemoto H, Konno S, Sugimoto H, Nakazora H, Nomoto N, Murata M, Kitazono H, Fujioka T (2011) Anti-TNF therapy using etanercept suppresses degenerative and inflammatory changes in skeletal muscle of older SJL/J mice. Exp Mol Pathol 90(3):264–270 21324312 10.1016/j.yexmp.2011.02.003

[CR104] Nicoll R, Gerasimidis K, Forrest E (2022) The role of micronutrients in the pathogenesis of alcohol-related liver disease. Alcohol Alcohol 57(3):275–282 34491307 10.1093/alcalc/agab060

[CR105] Nikolic A (2024) Chronic stress: tissue-specific impact on energy metabolism and metabolic adaptation, Dissertation, Düsseldorf, Heinrich-Heine-Universität

[CR106] Nishikawa H, Kim SK, Asai A (2024) Body composition in chronic liver disease. Int J Mol Sci 25(2):964 38256036 10.3390/ijms25020964 PMC10815828

[CR107] Oh S, So R, Shida T, Matsuo T, Kim B, Akiyama K, Isobe T, Okamoto Y, Tanaka K, Shoda J (2017) High-intensity aerobic exercise improves both hepatic fat content and stiffness in sedentary obese men with nonalcoholic fatty liver disease. Sci Rep 7(1):43029 28223710 10.1038/srep43029 PMC5320441

[CR108] Ohtani N, Kawada N (2019) Role of the gut–liver axis in liver inflammation, fibrosis, and cancer: a special focus on the gut microbiota relationship. Hepatol Commun 3(4):456–470 30976737 10.1002/hep4.1331 PMC6442695

[CR109] Orange ST, Leslie J, Ross M, Mann DA, Wackerhage H (2023) The exercise IL-6 enigma in cancer. Trends Endocrinol Metab 34(11):749–763 37633799 10.1016/j.tem.2023.08.001

[CR110] Pacifico L, Perla FM, Chiesa C (2019) Sarcopenia and nonalcoholic fatty liver disease: a causal relationship. Hepatobiliary Surg Nutr 8(2):144 31098363 10.21037/hbsn.2018.11.11 PMC6503235

[CR111] Pereyra AS, McLaughlin KL, Buddo KA, Ellis JM (2023) Medium-chain fatty acid oxidation is independent of l-carnitine in liver and kidney but not in heart and skeletal muscle. Am J Physiol Gastrointest Liver Physiol 325(4):G287–G294 37461880 10.1152/ajpgi.00105.2023 PMC10642992

[CR112] Phillips SM, Chevalier S, Leidy HJ (2016) Protein “requirements” beyond the RDA: implications for optimizing health. Appl Physiol Nutr Metab 41(5):565–572 26960445 10.1139/apnm-2015-0550

[CR113] Plank LD, Gane EJ, Peng S, Muthu C, Mathur S, Gillanders L, McIlroy K, Donaghy AJ, McCall JL (2008) Nocturnal nutritional supplementation improves total body protein status of patients with liver cirrhosis: a randomized 12‐month trial. Hepatology 48(2):557–566 18627001 10.1002/hep.22367

[CR114] Plauth M, Bernal W, Dasarathy S, Merli M, Plank LD, Schütz T, Bischoff SC (2019) ESPEN guideline on clinical nutrition in liver disease. Clin Nutr 38(2):485–521 30712783 10.1016/j.clnu.2018.12.022 PMC6686849

[CR115] Ponziani FR, Picca A, Marzetti E, Calvani R, Conta G, Del Chierico F, Capuani G, Faccia M, Fianchi F, Funaro B (2021) Characterization of the gut‐liver‐muscle axis in cirrhotic patients with sarcopenia. Liver Int 41(6):1320–1334 33713524 10.1111/liv.14876

[CR116] Postic C, Girard J (2008) Contribution of de novo fatty acid synthesis to hepatic steatosis and insulin resistance: lessons from genetically engineered mice. J Clin Invest 118(3):829–838 18317565 10.1172/JCI34275 PMC2254980

[CR117] Prokopidis K, Giannos P, Kirwan R, Ispoglou T, Galli F, Witard OC, Triantafyllidis KK, Kechagias KS, Morwani-Mangnani J, Ticinesi A (2023) Impact of probiotics on muscle mass, muscle strength and lean mass: a systematic review and meta-analysis of randomized controlled trials. J Cachexia Sarcopenia Muscle 14(1):30–44 36414567 10.1002/jcsm.13132 PMC9891957

[CR118] Rakoubian A, Khinchin J, Yarbro J, Kobayashi S, Liang Q (2025) Isoform-specific roles of AMP-activated protein kinase (AMPK) in cardiac physiology and pathophysiology. Front Cardiovasc Med 12:1638515 40860364 10.3389/fcvm.2025.1638515 PMC12370717

[CR119] Ramos-Tovar E, Muriel P (2020) Molecular mechanisms that link oxidative stress, inflammation, and fibrosis in the liver. Antioxidants 9(12):1279 33333846 10.3390/antiox9121279 PMC7765317

[CR120] Ranjbarvaziri S, Kooiker KB, Ellenberger M, Fajardo G, Zhao M, Vander Roest AS, Woldeyes RA, Koyano TT, Fong R, Ma N (2021) Altered cardiac energetics and mitochondrial dysfunction in hypertrophic cardiomyopathy. Circulation 144(21):1714–1731 34672721 10.1161/CIRCULATIONAHA.121.053575 PMC8608736

[CR121] Rashidi H, Luu N-T, Alwahsh SM, Ginai M, Alhaque S, Dong H, Tomaz RA, Vernay B, Vigneswara V, Hallett JM (2018) 3D human liver tissue from pluripotent stem cells displays stable phenotype in vitro and supports compromised liver function in vivo. Arch Toxicol 92(10):3117–3129 30155720 10.1007/s00204-018-2280-2 PMC6132688

[CR122] Refaat B, Abdelghany AH, Ahmad J, Abdalla OM, Elshopakey GE, Idris S, El‐Boshy M (2022) Vitamin D3 enhances the effects of omega‐3 oils against metabolic dysfunction‐associated fatty liver disease in rat. Biofactors 48(2):498–513 34767670 10.1002/biof.1804

[CR123] Roh E, Hwang SY, Yoo HJ, Baik SH, Lee J-H, Son SJ, Kim HJ, Park YS, Lee S-G, Cho BL (2022) Impact of non-alcoholic fatty liver disease on the risk of sarcopenia: a nationwide multicenter prospective study. Hepatol Int 16(3):545–554 34780030 10.1007/s12072-021-10258-8

[CR124] Romijn J, Pijl H (2009) The muscle–liver axis: does aerobic fitness induce intrahepatic protection against non-alcoholic fatty liver disease? J Physiol 587(Pt 8):1637 19369505 10.1113/jphysiol.2009.171868 PMC2683951

[CR125] Rondanelli M, Nichetti M, Peroni G, Faliva MA, Naso M, Gasparri C, Perna S, Oberto L, Di Paolo E, Riva A (2021) Where to find leucine in food and how to feed elderly with sarcopenia in order to counteract loss of muscle mass: practical advice. Front Nutr 7:622391 33585538 10.3389/fnut.2020.622391 PMC7874106

[CR126] Ruiz-Margáin A, Pohlmann A, Lanzerath S, Langheinrich M, Campos-Murguía A, Román-Calleja BM, Schierwagen R, Klein S, Uschner FE, Brol MJ (2023) Myostatin is associated with the presence and development of acute-on-chronic liver failure. JHEP Rep 5(8):100761 37554924 10.1016/j.jhepr.2023.100761 PMC10405090

[CR127] Saklayen MG (2018) The global epidemic of the metabolic syndrome. Curr Hypertens Rep 20(2):1–8 29480368 10.1007/s11906-018-0812-z PMC5866840

[CR128] Sartori R, Milan G, Patron M, Mammucari C, Blaauw B, Abraham R, Sandri M (2009) Smad2 and 3 transcription factors control muscle mass in adulthood. Am J Physiol Cell Physiol 296(6):C1248–C1257 19357234 10.1152/ajpcell.00104.2009

[CR129] Sartori R, Romanello V, Sandri M (2021) Mechanisms of muscle atrophy and hypertrophy: implications in health and disease. Nat Commun 12(1):330 33436614 10.1038/s41467-020-20123-1 PMC7803748

[CR130] Schumacher SM, Naga Prasad SV (2018) Tumor necrosis factor-α in heart failure: an updated review. Curr Cardiol Rep 20(11):117 30259192 10.1007/s11886-018-1067-7 PMC6311126

[CR131] Schwarz M, Meyer CE, Löser A, Lossow K, Hackler J, Ott C, Jäger S, Mohr I, Eklund EA, Patel AA (2023) Excessive copper impairs intrahepatocyte trafficking and secretion of selenoprotein P. Nat Commun 14(1):3479 37311819 10.1038/s41467-023-39245-3 PMC10264388

[CR132] Shaikh S, Ahmad K, Lim JH, Ahmad SS, Lee EJ, Choi I (2025) Skeletal muscle aging: enhancing skeletal muscle integrity and function as a potential pharmacological approach. Pharmaceuticals (Basel) 18(9):1407 41011274 10.3390/ph18091407 PMC12473867

[CR133] Sharpton SR, Maraj B, Harding-Theobald E, Vittinghoff E, Terrault NA (2019) Gut microbiome–targeted therapies in nonalcoholic fatty liver disease: a systematic review, meta-analysis, and meta-regression. Am J Clin Nutr 110(1):139–149 31124558 10.1093/ajcn/nqz042 PMC6599739

[CR134] Shen F, Zheng R-D, Sun X-Q, Ding W-J, Wang X-Y, Fan J-G (2017) Gut microbiota dysbiosis in patients with non-alcoholic fatty liver disease. Hepatobiliary Pancreat Dis Int 16(4):375–381 28823367 10.1016/S1499-3872(17)60019-5

[CR135] Shin C (2021) Effects of aerobic exercise and diet control on myonectin and fatty acid transporters in skeletal muscle and liver obese mice [Master’s Thesis]. Seoul: Seoul National University

[CR136] Shirakami Y, Kato J, Maeda T, Ideta T, Imai K, Sakai H, Shiraki M, Shimizu M (2023) Skeletal muscle atrophy is exacerbated by steatotic and fibrotic liver-derived TNF-α in senescence-accelerated mice. J Gastroenterol Hepatol 38(5):800–808 36890117 10.1111/jgh.16171

[CR137] Simon L, Bourgeois BL, Molina PE (2023) Alcohol and skeletal muscle in health and disease. Alcohol Res Curr Rev 43(1):04 10.35946/arcr.v43.1.04 PMC10627576 37937295

[CR138] Sinclair M, Grossmann M, Hoermann R, Angus PW, Gow PJ (2016) Testosterone therapy increases muscle mass in men with cirrhosis and low testosterone: a randomised controlled trial. J Hepatol 65(5):906–913 27312945 10.1016/j.jhep.2016.06.007

[CR139] Sinn DH, Kang D, Kang M, Guallar E, Hong YS, Lee KH, Park J, Cho J, Gwak GY (2022) Nonalcoholic fatty liver disease and accelerated loss of skeletal muscle mass: a longitudinal cohort study. Hepatology 76(6):1746–1754 35588190 10.1002/hep.32578

[CR140] Smiles WJ, Ovens AJ, Kemp BE, Galic S, Petersen J, Oakhill JS (2024) New developments in AMPK and mTORC1 cross-talk. Essays Biochem 68(3):321 38994736 10.1042/EBC20240007 PMC12055038

[CR141] Sokolove J, Strand V, Greenberg JD, Curtis JR, Kavanaugh A, Kremer JM, Anofrei A, Reed G, Calabrese L, Hooper M (2010) Risk of elevated liver enzymes associated with TNF inhibitor utilisation in patients with rheumatoid arthritis. Ann Rheum Dis 69(9):1612–1617 20448284 10.1136/ard.2009.112136

[CR142] Soleimani D, Ranjbar G, Rezvani R, Goshayeshi L, Razmpour F, Nematy M (2019) Dietary patterns in relation to hepatic fibrosis among patients with nonalcoholic fatty liver disease. Diabetes Metab Syndr Obes. 10.2147/dmso.s198744 30881075 10.2147/DMSO.S198744 PMC6420105

[CR143] Song F, Mao Y-J, Hu Y, Zhao S-S, Wang R, Wu W-Y, Li G-R, Wang Y, Li G (2022) Acacetin attenuates diabetes-induced cardiomyopathy by inhibiting oxidative stress and energy metabolism via PPAR-α/AMPK pathway. Eur J Pharmacol 922:174916 35341782 10.1016/j.ejphar.2022.174916

[CR144] Soykurt SÇ, Tekdemir SN (2024) Intermittent fasting and its potential effects on health. Cyprus J Med Sci 2024;9(4):221–227. 10.4274/cjms.2024.2023-109

[CR145] Steensberg A, Keller C, Starkie RL, Osada T, Febbraio MA, Pedersen BK (2002) IL-6 and TNF-α expression in, and release from, contracting human skeletal muscle. Am J Physiol Endocrinol Metab 283(6):E1272–E1278 12388119 10.1152/ajpendo.00255.2002

[CR146] Stine JG, Soriano C, Schreibman I, Rivas G, Hummer B, Yoo E, Schmitz K, Sciamanna C (2021) Breaking down barriers to physical activity in patients with nonalcoholic fatty liver disease. Dig Dis Sci 66(10):3604–3611 33098023 10.1007/s10620-020-06673-w PMC10321307

[CR147] Sullivan S, Kirk EP, Mittendorfer B, Patterson BW, Klein S (2012) Randomized trial of exercise effect on intrahepatic triglyceride content and lipid kinetics in nonalcoholic fatty liver disease. Hepatology 55(6):1738–1745 22213436 10.1002/hep.25548 PMC3337888

[CR148] Sun M, Chen M, Dawood F, Zurawska U, Li JY, Parker T, Kassiri Z, Kirshenbaum LA, Arnold M, Khokha R (2007) Tumor necrosis factor-α mediates cardiac remodeling and ventricular dysfunction after pressure overload state. Circulation 115(11):1398–1407 17353445 10.1161/CIRCULATIONAHA.106.643585

[CR149] Sun H, Sherrier M, Li H (2021) Skeletal muscle and bone–emerging targets of fibroblast growth factor-21. Front Physiol 12:625287 33762965 10.3389/fphys.2021.625287 PMC7982600

[CR150] Szukiewicz D (2023) Molecular mechanisms for the vicious cycle between insulin resistance and the inflammatory response in obesity. Int J Mol Sci 24(12):9818 37372966 10.3390/ijms24129818 PMC10298329

[CR151] Takahashi A, Abe K, Usami K, Imaizumi H, Hayashi M, Okai K, Kanno Y, Tanji N, Watanabe H, Ohira H (2015) Simple resistance exercise helps patients with non-alcoholic fatty liver disease. Int J Sports Med 94(10):848–852 10.1055/s-0035-1549853 26090879

[CR152] Tandon P, Ismond KP, Riess K, Duarte-Rojo A, Al-Judaibi B, Dunn MA, Holman J, Howes N, Haykowsky MJF, Josbeno DA (2018) Exercise in cirrhosis: translating evidence and experience to practice. J Hepatol 69(5):1164–1177 29964066 10.1016/j.jhep.2018.06.017

[CR153] Tang K-T, Dufour J-F, Chen P-H, Hernaez R, Hutfless S (2020) Antitumour necrosis factor-α agents and development of new-onset cirrhosis or non-alcoholic fatty liver disease: a retrospective cohort. BMJ Open Gastroenterol. 10.1136/bmjgast-2019-000349 32377366 10.1136/bmjgast-2019-000349 PMC7199652

[CR154] Tarantino G, Citro V, Balsano C (2021) Liver-spleen axis in nonalcoholic fatty liver disease. Expert Rev Gastroenterol Hepatol 15(7):759–769 33878988 10.1080/17474124.2021.1914587

[CR155] Thorp A, Stine JG (2020) Exercise as medicine: the impact of exercise training on nonalcoholic fatty liver disease. Curr Hepatol Rep 19(4):402–411 33767944 10.1007/s11901-020-00543-9 PMC7987125

[CR156] Tian S, Lei Y, Zhao F, Che J, Wu Y, Lei P, Kang YE, Shan Y (2024) Improving insulin resistance by sulforaphane via activating the Bacteroides and Lactobacillus SCFAs–GPR–GLP1 signal axis. Food Funct 15(17):8644–8660 39045769 10.1039/d4fo01059k

[CR157] Topan M-M, Sporea I, Dănilă M, Popescu A, Ghiuchici A-M, Lupuşoru R, Şirli R (2021) Impact of sarcopenia on survival and clinical outcomes in patients with liver cirrhosis. Front Nutr 8:766451 34746216 10.3389/fnut.2021.766451 PMC8566695

[CR158] Tripathi A, Debelius J, Brenner DA, Karin M, Loomba R, Schnabl B, Knight R (2018) The gut–liver axis and the intersection with the microbiome. Nat Rev Gastroenterol Hepatol 15(7):397–411 29748586 10.1038/s41575-018-0011-z PMC6319369

[CR159] Tsien C, Davuluri G, Singh D, Allawy A, Ten Have GA, Thapaliya S, Schulze JM, Barnes D, McCullough AJ, Engelen MP (2015) Metabolic and molecular responses to leucine‐enriched branched chain amino acid supplementation in the skeletal muscle of alcoholic cirrhosis. Hepatology 61(6):2018–2029 25613922 10.1002/hep.27717 PMC4441611

[CR160] Usui S, Takashima S, Goten C, Inoue O, Takeda Y, Yamaguchi K, Hashimuko D, Takamura M (2023) Hepatokine selenoprotein P has an important role in cardiac remodeling. Eur Heart J 44(Supplement_2):ehad655. 3152

[CR161] Vachliotis ID, Polyzos SA (2023) The role of tumor necrosis factor-alpha in the pathogenesis and treatment of nonalcoholic fatty liver disease. Curr Obes Rep 12(3):191–206 37407724 10.1007/s13679-023-00519-y PMC10482776

[CR162] Vakilpour A, Amini-Salehi E, Soltani Moghadam A, Keivanlou M-H, Letafatkar N, Habibi A, Hashemi M, Eslami N, Zare R, Norouzi N (2024) The effects of gut microbiome manipulation on glycemic indices in patients with non-alcoholic fatty liver disease: a comprehensive umbrella review. Nutr Diabetes 14(1):25 38729941 10.1038/s41387-024-00281-7 PMC11087547

[CR163] Vuille-Lessard É, Lange N, Riebensahm C, Dufour J-F, Berzigotti A (2021) Dietary interventions in liver diseases: focus on MAFLD and cirrhosis. Curr Hepatol Rep 20(2):61–76

[CR164] Wallace MA, Aguirre NW, Marcotte GR, Marshall AG, Baehr LM, Hughes DC, Hamilton KL, Roberts MN, Lopez‐Dominguez JA, Miller BF (2021) The ketogenic diet preserves skeletal muscle with aging in mice. Aging Cell 20(4):e13322 33675103 10.1111/acel.13322 PMC8045940

[CR165] Wang G-Y, Zhang X-Y, Wang C-J, Guan Y-F (2023) Emerging novel targets for nonalcoholic fatty liver disease treatment: evidence from recent basic studies. World J Gastroenterol 29(1):75 36683713 10.3748/wjg.v29.i1.75 PMC9850950

[CR166] Willcockson H, Greco A, Fragassi A, Palomba R, Kwon K, Ozkan H, Bartlett ST, Loeser RF, Decuzzi P, Longobardi L (2025) Sustained release of exogeneous fetuin-A from Hyaluronic acid microplates decreases joint degeneration, synovial hyperplasia and muscle damage in a murine post-traumatic osteoarthritis model. Arthritis Res Ther 27(1):178 40999539 10.1186/s13075-025-03636-2 PMC12465764

[CR167] Wong R, Yuan L-Y (2024) Sarcopenia and metabolic dysfunction associated steatotic liver disease: time to address both. World J Hepatol 16(6):871 38948439 10.4254/wjh.v16.i6.871 PMC11212657

[CR168] Xiong Y, Peng Q, Cao C, Xu Z, Zhang B (2021) Effect of different exercise methods on non-alcoholic fatty liver disease: a meta-analysis and meta-regression. Int J Environ Res Public Health 18(6):3242 33801028 10.3390/ijerph18063242 PMC8004001

[CR169] Yano N, Zhao YT, Zhao TC (2021) The physiological role of irisin in the regulation of muscle glucose homeostasis. Endocrines 2(3):266–283 35392577 10.3390/endocrines2030025 PMC8986094

[CR170] Youm Y-H, Nguyen KY, Grant RW, Goldberg EL, Bodogai M, Kim D, D’agostino D, Planavsky N, Lupfer C, Kanneganti TD (2015) The ketone metabolite β-hydroxybutyrate blocks NLRP3 inflammasome–mediated inflammatory disease. Nat Med 21(3):263–269 25686106 10.1038/nm.3804 PMC4352123

[CR171] Zelber-Sagi S, Buch A, Yeshua H, Vaisman N, Webb M, Harari G, Kis O, Fliss-Isakov N, Izkhakov E, Halpern Z (2014) Effect of resistance training on non-alcoholic fatty-liver disease a randomized-clinical trial. World J Gastroenterol 20(15):4382 24764677 10.3748/wjg.v20.i15.4382 PMC3989975

[CR172] Zhang C, Liu S, Yang M (2023) Treatment of liver fibrosis: past, current, and future. World J Hepatol 15:755–774 37397931 10.4254/wjh.v15.i6.755 PMC10308286

[CR173] Zhang J, Hu Z, Horta CA, Yang J (2023) Regulation of epithelial-mesenchymal transition by tumor microenvironmental signals and its implication in cancer therapeutics. Semin Cancer Biol. 10.1016/j.semcancer.2022.12.002 36521737 10.1016/j.semcancer.2022.12.002 PMC10237282

[CR174] Zhang F, Wang Z, Zhang H, Zuo Y (2025) Metabolic dysfunction-associated steatotic liver disease (MASLD) attenuates the predictive value of the triglyceride–glucose index for carotid plaque: evidence of insulin resistance-independent pathways. Front Endocrinol 16:1696652 10.3389/fendo.2025.1696652 PMC12571656 41180199

[CR175] Zhao Y, Zhou Y, Wang D, Huang Z, Xiao X, Zheng Q, Li S, Long D, Feng L (2023) Mitochondrial dysfunction in metabolic dysfunction fatty liver disease (MAFLD). Int J Mol Sci 24(24):17514 38139341 10.3390/ijms242417514 PMC10743953

[CR176] Zhao C, Wu Y, Zhu S, Liu H, Xu S (2024) Irisin protects musculoskeletal homeostasis via a mitochondrial quality control mechanism. Int J Mol Sci 25(18):10116 39337601 10.3390/ijms251810116 PMC11431940

[CR177] Zhou Q, Hu H, Hu L, Liu S, Chen J, Tong S (2024) Association between processed and unprocessed red meat consumption and risk of nonalcoholic fatty liver disease: a systematic review and dose-response meta-analysis. J Glob Health 14:04060 38665062 10.7189/jogh.14.04060 PMC11046257

